# Ribonucleosides from tRNA in hyperglycemic mammalian cells and diabetic murine cardiac models

**DOI:** 10.1016/j.lfs.2023.121462

**Published:** 2023-02-02

**Authors:** Taylor A. Dodson, Stephan Nieuwoudt, Chase N. Morse, Valinteshley Pierre, Chao Liu, Samuel E. Senyo, Erin G. Prestwich

**Affiliations:** a Department of Medicinal and Biological Chemistry, College of Pharmacy and Pharmaceutical Sciences, University of Toledo, Toledo, OH, United States; b Department of Biomedical Engineering, School of Engineering, Case Western Reserve University, Cleveland, OH, United States

**Keywords:** Mass spectrometry, tRNA, Diabetes, Cardiac, Hyperglycemia

## Abstract

**Aims:**

Cardiomyopathy is a diabetic comorbidity with few molecular targets. To address this, we evaluated transfer RNA (tRNA) modifications in the diabetic heart because tRNA modifications have been implicated in diabetic etiologies.

**Main methods:**

tRNA was isolated from aorta, apex, and atrial tissue of healthy and diabetic murine hearts and related hyperglycemic cell models. tRNA modifications and canonical ribonucleosides were quantified by liquid-chromatography tandem mass spectrometry (LC-MS/MS) using stable isotope dilution. Correlations between ribonucleosides and diabetic comorbidity pathology were assessed using statistical analyses.

**Key findings:**

Total tRNA ribonucleoside levels were analyzed from cell types and healthy and diabetic murine heart tissue. Each heart structure had characteristic ribonucleoside profiles and quantities. Several ribonucleosides were observed as significantly different in hyperglycemic cells and diabetic tissues. In hyperglycemic models, ribonucleosides N^4^-acetylcytidine (ac^4^C), 5-methoxycarbonylmethyl-2-thiouridine (mcm^5^s^2^U), 5-methylcytidine (m^5^C), and N^1^-methylguanosine (m^1^G) were anomalous. Specific tRNA modifications known to be on murine tRNA^Ini(CAU)^ were higher in diabetic heart tissue which suggests that tRNA modifications could be regulating translation in diabetes.

**Significance:**

We identified tRNA ribonucleosides and tRNA species associated with hyperglycemia and diabetic etiology.

## 1. Introduction

The building blocks of RNA are canonical ribonucleosides: cytidine (rC), guanosine (rG), adenosine (rA), and uridine (rU). RNA from all domains of life contain ribonucleoside chemical modifications such as enzymatic methylations, acetylations, and thiolations [1,2]. Though all types of RNA are known to be modified, tRNAs are the most extensively modified type of RNA. Human diseases such as cancer or diabetes can be caused by aberrations to tRNA modifications [3–8]. For example, the impaired function of Cdkal1, an enzyme which catalyzes the methylthiolation of N^6^-threonylcarbamoyladenosine (t^6^A), due to iron-deficiency can lead to mistranslation and processing of proinsulin, causing diabetes in mice [3]. Additionally, cellular anomalies to endogenous modifications such as 5-methoxycarbonylmethyl-2-thiouri-dine (mcm^5^s^2^U) [9], 5-carbamoylmethyluridine (ncm^5^U) [10], 5-methylcytidine (m^5^C) [11], and N^4^-acetylcytidine (ac^4^C) [12], are associated with oxidative stress-induced impairment of tRNA modification enzymes, thereby potentially affecting translational control. Identification and characterization of tRNA modification regulatory roles are important in understanding disease etiology at the molecular level.

The incidence of heart failure is elevated in people with diabetes independent of obesity, hypertension, dyslipidemia, and coronary heart disease [13,14]. Many antidiabetic drugs target systemic hyperglycemia without reducing risk of heart disease [15]. Atherosclerosis, fibrosis, and maladaptive hypertrophy represent abnormal phenotypes of distinct cell types that manifest as heart disease during diabetes [16]. Thus, evaluating diabetic impact at the cellular scale is critical to understanding the diverse clinical presentations of diabetic heart disease [16].

Modified tRNA ribonucleosides from *in vitro* and *in vivo* samples were analyzed to assess hyperglycemic reprogramming of tRNA modifications. The results of this study will help elucidate the association of tRNA modifications and diabetic cardiac comorbidities.

## 2. Materials and methods

### 2.1. Maintenance of mammalian cell cultures in high or low glucose

MEK, 3T3, and H9C2 cells were grown and harvested using the same protocol. Cells were grown in 1× DMEM (ThermoFisher, Waltham, Massachusetts) with 10 % FBS (*Pkd1*^*del34/del34*^ MEK) or 2.5 % FBS (HyClone Laboratories Inc., Logan, Utah) (CRL-1446 H9C2 and CRL-1658 3T3), and 1 % Penicillin/Streptomycin (SV30010, Hyclone Laboratories Inc., Logan, Utah) at 37 °C, 5 % CO_2_. When cells were 70–80 % confluent, they were washed with 1× phosphate-buffered saline (PBS), trypsinized, and passaged with fresh 1× DMEM media into either 1× DMEM high glucose (4.5 g/L d-Glucose) or 1× DMEM low glucose (1 g/L d-Glucose). The cells were cultured for 4 days in either low or high glucose conditions. Biological replicates are defined as cells split from a frozen cell stock and passaged at least two times. Treated cells were trypsinized, washed with PBS, flash frozen in liquid nitrogen, and stored in −80 °C freezers until analysis. There are four MEK biological replicates per cell treatment, and five H9C2 and 3T3 biological replicates per cell treatment, respectively.

### 2.2. Mouse models

Healthy female C57BL/6N mice were compared to an established type II diabetes model induced by streptozotocin drug in combination with a high fat diet [17,18]. Young adult (3–4 week old) healthy control mice (*n* = 4) were fed a low fat diet (10 % Kcal % fat) throughout the duration of the study. The diabetic group (*n* = 4) of the same age were fed a high fat diet (60 % Kcal % fat) for the duration of the study. Streptozotocin was administered to the 7-week-old diabetic mouse group for 5 consecutive days (40 mg/kg daily intraperitoneal injections) to induce pancreatic β-cell failure. Mice achieved a fasting blood glucose level (>250 mg/dL) by 10 days post-streptozotocin determined by glucometer measurements by tail snip. The established combination of high fat diet and streptozotocin achieves a type II diabetes phenotype of impaired glucose handling. For the duration of the study, glucose levels were confirmed with the glucose tolerance test at weeks 6, 9, and 12 after 6-hour fasts, respectively (Supplementary Fig. S1). Glucose tolerance was assayed after intraperitoneal injection of dextrose (0.25 mL max volume) into fasting mice at 2 mg/g body weight (~15–20 g) which approximates to 100 mg/mL injection. Blood samples were collected at 0, 15, 30, 60, and 120 min from the tail vein (repeated sampling after snip) and used for blood glucose measurement. All animals were euthanized by CO_2_ asphyxiation and organs harvested at 12 weeks (16-week-old mice) [17]. Organs were stored at −80 °C until nucleic acid isolation was performed. The mouse studies were performed in accordance with protocols approved by CWRU.

To address cardiac function, mice were anesthetized by 5 % isoflurane (Patterson veterinary, Greeley, CO, USA) and maintained at 1 % to 1.5 % isoflurane as previously published [19]. Body temperature was maintained at 37 °C and heart rate was kept at 400 to 500 beats per minute. M-mode and B-mode were recorded along the long axis using a Vevo 3100 ultrasound imaging system (VisualSonics) equipped with a MX550D transducer. Cardiac function was analyzed using Vevo lab (VisualSonics) at 12 weeks (Supplementary Fig. S2).

### 2.3. Pseudomonas aeruginosa growth in ^13^C labeled glucose medium

*P. aeruginosa* PA14 was grown in M9 minimal media (2 mM MgSO_4_, 0.1 mM CaCl_2_, 0.4 % weight/volume (^*w*^/_*v*_) glucose, 47.9 mM Na_2_HPO_4_, 22 mM KH_2_PO_4_, 8.2 mM NaCl, 18.7 mM NH_4_Cl), with the only carbon source being d-glucose-^13^C_6_, (Sigma-Aldrich, Munich, Germany). To ensure all carbons were isotopically labeled (^13^C), cells were maintained in M9 minimal media containing ^13^C labeled glucose for at least two passages. *P. aeruginosa* was grown to OD_600_ ≥ 1.5 at 37 °C and cells were harvested by centrifugation and washed 1× with 0.9 % saline.

### 2.4. Isolation of total RNA

Total nucleic acid was isolated from *P. aeruginosa* cells, mammalian cultures, and tissues utilizing an RNA isolation method [20] which was modified. About 100–500 mg of cells or tissue were lysed at 65 °C with 1 mL buffer (2 % ^*w*^/_*v*_ hexadecyltrimethylammonium bromide (Sigma-Aldrich), 100 mM Tris-HCl pH 8 (Fisher Scientific), 2 M NaCl (Fisher Chemical), 20 mM ethylenediamine tetraacetic acid, disodium salt dihydrate pH 8 (Fisher Chemical)). At the cell lysis step, 5–10 mg of poly (vinylpolypyrrolidone) (Sigma-Aldrich) and 30 μL 2-mercaptoethanol (VWR Life Science) were added per 1 mL of lysate. An equal volume of 24:1 chloroform (Fisher Chemical, Hampton, New Hampshire): isoamyl alcohol (Bio Basic) was added to the cell lysate, vortexed, and centrifuged. The 24:1 chloroform: isoamyl alcohol extraction was repeated once with the aqueous layer. The resulting aqueous layer was transferred to a new tube, and total nucleic acid was precipitated with ≥2 volumes of 100 % ethanol, then centrifuged. The nucleic acid pellet was solubilized in 500 μL Milli-Q nuclease-free H_2_O. For nuclease-free H_2_O, Milli-Q H_2_O was treated with 0.1 % diethylpyrocarbonate (Millipore Sigma), then autoclaved. The solubilized nucleic acid was incubated at 50 °C with 300 mg proteinase K (Sigma-Aldrich) to digest proteins, and then extracted with 25:24:1 phenol: chloroform: isoamyl alcohol pH 6.7 (Fisher Scientific). Two chloroform extractions were performed to remove residual phenol in the aqueous layer. To remove polysaccharides, an equal volume of diethyl ether was added to the aqueous layer and centrifuged [21]. The resulting aqueous layer was transferred to a new tube and total nucleic acid was precipitated with ≥2 volumes 100 % ethanol, and subsequently centrifuged. Total nucleic acid was solubilized in Milli-Q nuclease-free H_2_O and quantified via spectrophotometer. Total RNA from mammalian cells and tissues were isolated the same way except tissues were homogenized in the lysis buffer. Also, for mammalian cells and tissue, poly(vinylpolypyrrolidone) (Sigma-Aldrich) was not added, and the diethyl ether (Sigma-Aldrich) extraction was not performed.

### 2.5. Isolation and collection of isotopically labeled RNA nucleosides

In 50 μL volumes, total nucleic acid (50–100 μg) was enzymatically digested to nucleosides adapted from a previously reported procedure [22] (2 mM MgCl_2_ (Fisher Scientific), 40 mM Tris (pH 8), 4 U Benzonase (Millipore Sigma), 2 U DNase I (Millipore Sigma), 0.1 U phosphodiesterase (US Biologicals), 10 U alkaline phosphatase (Sigma-Aldrich), 50 ng RNase A (Sigma-Aldrich), and 18.6 μM pentostatin (adenosine deaminase inhibitor) (Sigma-Aldrich)) and incubated overnight at 37 °C. After incubation, the digest was filtered through 10 kDa centrifuge filters (Pall Life Sciences) to remove enzymes. After filtration, the samples were dried under vacuum and resolubilized in 50 μL Milli-Q nuclease-free H_2_O.

RNA and DNA canonical nucleosides were separated from modified nucleosides using high-performance liquid chromatography (HPLC). The Varian Microsorb-MV C18 column 250 × 4.6 mm was utilized for HPLC separations at 30 °C. The solvents were 5 mM ammonium acetate (solvent A) and acetonitrile (solvent B) with a flow rate of 0.5 mL/min. The elution began with 100 % solvent A for 25 min, increased to 1 % B (25 min–40 min), followed by step gradients of 1 % B (40 min–65 min), 2 % B (65 min–75 min), then 3 % B (75 min–85 min). Then a gradient from 3 to 14 % B (85 min–120 min), then 14–60 % B (120 min–135 min). A final wash step at 98 % B was performed and then re-equilibration at 100 % A for 30 min. 50 μg of enzymatically digested ^13^C isotopically labeled nucleosides were injected onto the column. All of the eluent other than RNA and DNA canonical nucleosides were fraction collected, combined, dried, and solubilized in Milli-Q nuclease-free H_2_O (Supplementary Fig. S3). RNA and DNA canonical nucleosides were visible by UV, and were fraction collected separately.

### 2.6. tRNA isolation from mammalian cells and tissue

tRNA was isolated from total nucleic acid by PAGE. Either a 15 % or 20 % denaturing (urea) PAGE was utilized to separate nucleic acid species. The RiboRuler Low Range RNA Ladder (ThermoFisher, Waltham, Massachusetts) or DynaMarker, Small RNA II Ladder (DiagnoCine, Hackensack, New Jersey) was used as a size reference. The gels were stained with ethidium bromide to visualize nucleic acid. Nucleic acid species below 100 nucleotides (nts) and above 50 nts was deemed tRNA, similarly to other work [23]. tRNA was excised from the gel and isolated by crush and soak method [24]. The resulting tRNA was quantified via spectrophotometer. Approximately 50 pmol (roughly 1.3 μg) of tRNA was digested to nucleosides via the enzymatic digest, filtered, dried, and resolubilized as described above.

### 2.7. LC-MS/MS separation and quantitation of tRNA nucleosides

#### 2.7.1. LC-MS/MS method

Ribonucleoside standards were purchased from Toronto Research Chemicals (Ontario, Canada), Carbosynth (San Diego, CA), Ark Pharm, Inc. (Arlington Heights, IL), or Sigma-Aldrich (St. Louis, MO) as defined in Supplementary Table S1. The tRNA samples were analyzed using a Shimadzu Nexera XR LC-20-AD HPLC equipped with a photodiode array detector (PDA) and a triple quadrupole mass spectrometer Shimadzu 8050 (Japan). A Phenomenex Luna Omega Polar C18 column was used (1.6 μm particle size, 100 Å pore size, 150 × 2.1 mm) at 21 °C and with a 0.17 mL/min flow rate. Solvents consisted of 0.1 % formic acid in Millipore Q water (solvent A) and 0.1 % formic acid in acetonitrile (solvent B). The elution began at 100 % A for 40 min, then linear gradients followed: 0 %–5 % B 40 min–80 min; 5 %–6 % B 80 min–100 min; 6 %–70 % B 100 min–120 min. The column was washed with 98 % B and reequilibrated at 100 % A for 30 min (Supplementary Fig. S4). The RNA canonical nucleosides were quantified via PDA and then diverted to waste, while all of the modified nucleosides were detected and quantified via MS with an electrospray ion (ESI) source. The ESI parameters were: DL temperature 125 °C, CID gas 270 kPa, nebulizing gas flow 3.0 L/min, drying gas flow 17.0 L/min, heating gas flow 3.0 L/min, interface temperature 300 °C, heat block temperature 400 °C, and interface voltage 4 kV. Samples were analyzed in positive ion mode in multiple reaction monitoring (MRM) mode. Ribonucleosides generally fragmented at the glycosidic bond, causing the neutral loss of the ribose or 2^′^-*O*-methylribose (Supplementary Table S1). Pseudouridine and methylated pseudouridine ribonucleosides fragmented in a unique pattern (Supplementary Fig. S5). RNA modifications were identified by their MRM and their retention times.

#### 2.7.2. Data acquisition and analysis

Approximately 1.2 pmol of ^13^C labeled ribonucleoside modifications collected fractions and 10.0 pmol of total digested tRNA sample (about 0.26 μg ^12^C total tRNA) were injected for LC-MS/MS analysis. Each sample containing ^13^C labeled standards and endogenous tRNA ribonucleosides were injected onto the column at 10 μL injection volumes. MRMs of ^13^C isotopically labeled ribonucleosides were as follows: m^6,6^A, *m*/*z* 308 > 171; t^6^A, *m*/*z* 428 > 291; m^2^A; *m*/*z* 293 > 156; Am, *m*/*z* 293 > 141; m^6^A, *m*/*z* 293 > 156; i^6^A, *m*/*z* 351 > 214; m^5^C, *m*/*z* 268 > 131; s^2^C, *m*/*z* 269 > 132; Cm, *m*/*z* 268 > 116; m^4^Cm, *m*/*z* 283 > 131; D, *m*/*z* 256 > 119; Ψ, *m*/*z* 254 > 218; m^5^U, *m*/*z* 269 > 132; s^4^U, *m*/*z* 270 > 133; m^3^U, *m*/*z* 269 > 132; Um, *m*/*z* 269 > 117; m^7^G, *m*/*z* 309 > 172; m^2^G, *m*/*z* 309 > 172; m^1^G, *m*/*z* 309 > 172; Gm, *m*/*z* 309 > 157; I, *m*/*z* 279 > 142. UV traces of canonical ribonucleosides and MS traces of modified ribonucleosides were extracted from Shimadzu LabSolutions Software using manual peak integration. Internal calibration (^13^C isotopically labeled ribonucleosides) and external calibration (calibration curves) were combined for quantification by division of MS signal of corresponding unlabeled (^12^C) and labeled (^13^C) ribonucleosides [25]. Any modified ribonucleosides without a respective ^13^C isotopically labeled standard were quantified by division of ^12^C MS signal over the total integrated sum of all ^13^C isotopically labeled ribonucleosides.

### 2.8. Statistics

Data represents the average of tRNA modification levels from five biological replicates (n) of 3T3 and H9C2 cells and 4 biological replicates of MEK cells for both high and low glucose. There were 3 biological replicates analyzed from each tissue type for both healthy and diabetic tissue. Student’s unpaired *t*-tests were performed to determine statistical significance between cells cultured in low versus high glucose media, and healthy versus diabetic tissue. Outliers were removed via Grubbs’ tests when there were >3 biological replicates (*n* > 3). To determine significantly different tRNA ribonucleoside levels in cell lines cultured in low glucose or healthy tissue, respectively, one-way ANOVA with Tukey’s test for multiple comparisons were performed. Hierarchical clustering analyses were performed using hclust function in R version 4.1 with method set to complete. Heat map dendrograms were created using the pheatmap (version 1.0.12) R package (RRID: SCR_016418) [26]. PCA was performed using Prism (version 9.4.0) from GraphPad. Values excluded from data by Grubbs’ test were treated as averages for principal component analysis (PCA).

## 3. Results

Hyperglycemia causes reactive oxygen species (ROS) [27], which have been shown to impact the activity and structures of proteins [28], DNA [29,30], and tRNA [9]. To determine the impact of high glucose levels to mammalian tRNA modification profiles, tRNA from *in vitro* mammalian cells and murine cardiac tissue were analyzed. Murine embryonic fibroblasts (3T3), rat embryonic cardiomyoblasts (H9C2), and murine embryonic kidney (MEK) cell lines were chosen because of their association with tissues affected by hyperglycemia [31]. The *in vitro* cells were passaged in high (4.5 g/L) or low (1 g/L) glucose conditions to mimic pathological hyperglycemic and low postprandial-glycemic levels, respectively. Cardiovascular disease is a common diabetic comorbidity [16,32–35], therefore murine aorta, ventricle (apex), and atrial heart tissues were compared across non-diabetic control mice and the induced diabetic mouse model.

An established model for type II diabetes was applied to C57BL/6N mice using a combination of high fat diet and streptozotocin drug. Glucose intolerance is first demonstrated at 6 weeks by a glucose tolerance test (Supplementary Fig. S1). Using echocardiography analysis at 9 weeks post-drug onset (week-12 of the study), these diabetic mice exhibit the earliest stages of cardiac dysfunction determined by trends of chamber dilation and reduced diastolic function (Supplementary Fig. S2). Of note, we did not observe cardiomyocyte hypertrophy by histological analysis (data not shown).

To analyze tRNA modifications, we developed and utilized methodologies outlined in Schematic 1. Total nucleic acid was isolated using a procedure adapted from other work [20], then separated via high percentage (15 % or 20 %) denaturing polyacrylamide gel electrophoresis (PAGE). tRNA was gel purified to ensure separation from other small RNAs (Supplementary Fig. S6) then enzymatically digested to nucleosides. Triple quadrupole MS/MS was utilized for quantitative targeted analysis of ribonucleosides in positive ion mode (Supplementary Fig. S4). For quantitative analysis of tRNA modified ribonucleosides, ^13^C isotopically labeled ribonucleoside standards were biosynthesized [25]. Canonical ribonucleosides were quantified via photodiode array (PDA) while ribonucleoside modifications were quantified by MS (Supplementary Fig. S4). This methodology was utilized for quantifying tRNA ribonucleoside levels in all cell and tissue models.

**Fig. 1.**
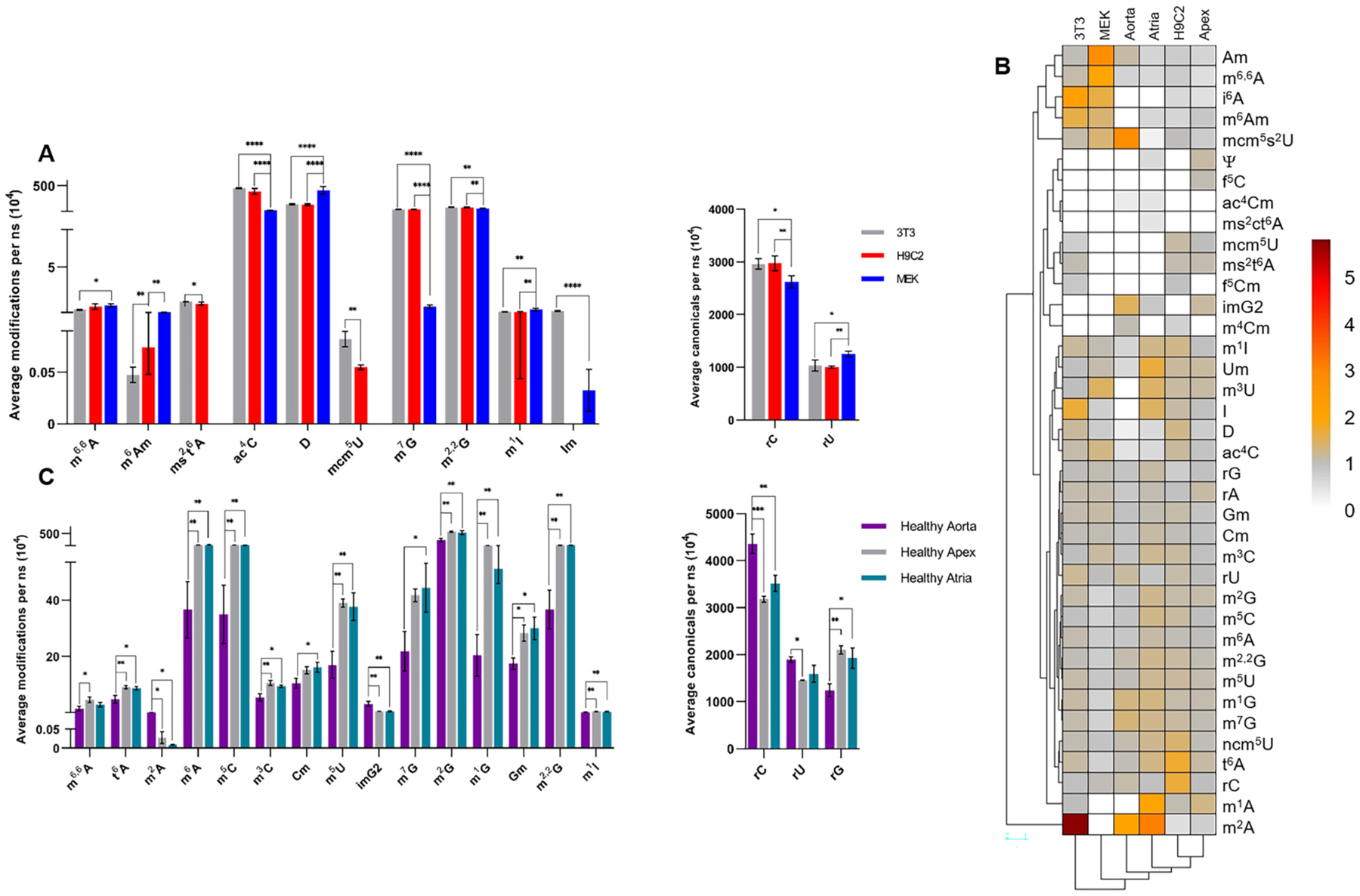
Comparison of tRNA ribonucleoside levels quantified via LC-MS/MS analysis: A) Graphical representation of significantly different tRNA ribonucleoside levels from *in vitro* cells grown in low glucose media; B) Hierarchical clustering of the average tRNA ribonucleoside level discovered by LC-MS/MS analysis presented as ratios of high glucose (HG) versus low glucose (LG) (*in vitro* cells) or diabetic versus healthy (*in vivo* murine tissue) (Supplementary Table S2). Red indicates higher ribonucleoside levels in HG cells or diabetic tissue, whereas gray indicates higher ribonucleoside levels in LG cells or healthy tissue. For boxes in the heat map to be above 0, values must be above the limit of detection (LOD) for both HG and LG, or healthy and diabetic. White boxes (or 0) indicate ribonucleoside level were below the LOD, or there were <3 biological replicates for cells or <2 biological replicates for tissue; C) Graphical representation of significantly different tRNA ribonucleoside levels from *in vivo* tissue from non-diabetic mice; One-way ANOVA was performed to determine significance, where *p* < 0.0001****, p 0.0001 < 0.001***, p 0.001 < 0.01**, and p 0.01 < 0.05* (*n* ≥ 3).

Many tRNA ribonucleoside levels were significantly different in 3T3, MEK, and H9C2 cells grown in several passages of low glucose media (Fig. 1A). These observations could reflect intrinsic differences in tRNA modifying enzyme activity. Variations to canonical and modified ribonucleoside abundance could also indicate changes in tRNA expression levels [36–38]. Here, total nucleic acid was analyzed via PAGE, and bands representing tRNA or multiple tRNA species were seemingly differentially abundant between the examined cell types (Supplementary Fig. S6). These findings indicate that the variations in tRNA modification levels between cell types could be due to tRNA expression levels.

Total tRNA ribonucleosides from cells cultured in high or low glucose media were analyzed. Principal component analysis (PCA) was performed to predict correlations between glucose levels, tRNA modification levels, and *in vitro* cell types (Supplementary Fig. S7). In response to ROS formation, Alkbh8, an enzyme required for the biosynthesis of 5-methyoxycarbonylmethyluridine (mcm^5^U) [10], is induced in embryonic fibroblasts [9]. Here, 3T3 cells passaged in high glucose, showed lower levels of mcm^5^U (Table 1). There was a correlation between 3T3 cells grown in low glucose media and the modification mcm^5^U (Supplementary Fig. S7). This contrasted with the higher levels of ac^4^C, rA, and N^6^-methyl-2^′^-*O*-methyladenosine (m^6^Am) in 3T3 cells grown in high glucose media (Table 1). These findings were confirmed via PCA as m^6^Am was inversely correlated with mcm^5^U. Additionally, rA and ac^4^C were correlated with 3T3 cells grown in high glucose media (Supplementary Fig. S7). The correlations associated with high glucose media were dependent upon cell type. For example, N^6^-isopentenyladenosine (i^6^A) and m^6^Am levels increased in 3T3 and MEK cells grown in high glucose media but decreased in H9C2 cells when cultured in high glucose media (Fig. 1B and Supplementary Table S2). These H9C2 cell hyperglycemic correlations could be explained by cardiomyocyte metabolism [39] or differences between mouse and rat tRNA [2]. Because fetal bovine serum (FBS) concentration differences can modulate molecular mechanisms in pluripotent stem cells [40], it is possible that differences in FBS concentration could cause the variations seen here between tRNA ribonucleosides in MEK cells and H9C2 or 3T3 cells.

We hypothesized that tRNA ribonucleoside trends in established diabetic versus non-diabetic mouse models could be glucose-dependent. Since the abundance of tRNA species (and likely tRNA modifications) are tissue specific [36], and tRNA modification variations were found in different parts of murine brains [23], we assessed whether different muscular heart structures exhibit diverse tRNA modification profiles. First, the aorta, apex, and atria of non-diabetic (healthy) mice were harvested for analysis. tRNA was analyzed using the methodologies outlined in Schematic 1. Levels of fifteen modified ribonucleosides and three canonical ribonucleosides were significantly distinct in different regions of the heart in non-diabetic mice (Fig. 1C). Since different cardiovascular structures are functionally and biologically distinct and can be differentially diseased (e.g., coronary artery disease, aortic aneurysms, pericardial disease, etc.), it is unsurprising that tRNA ribonucleoside modifications were different in heart structures. This is also an important observation for classification of disease states [4].

Total tRNA ribonucleoside levels were analyzed from healthy and diabetic murine aorta, apex, and atrial tissue (Supplementary Table S3). Several ribonucleosides were significantly different in hyperglycemic cells and diabetic tissues, including rA, N^1^-methylguanosine (m^1^G), m^6^Am, t^6^A, and 5-methylcytidine (m^5^C) compared to corresponding controls (Table 1). For example, levels of t^6^A in diabetic atrial tissue were similar to those in H9C2 cells (rat embryonic cardiomyoblasts) passaged in high glucose media. Amounts of t^6^A in the atria, aorta, and apex were all higher in diabetic mice versus healthy mice (Supplementary Table S3).

**Fig. 2.**
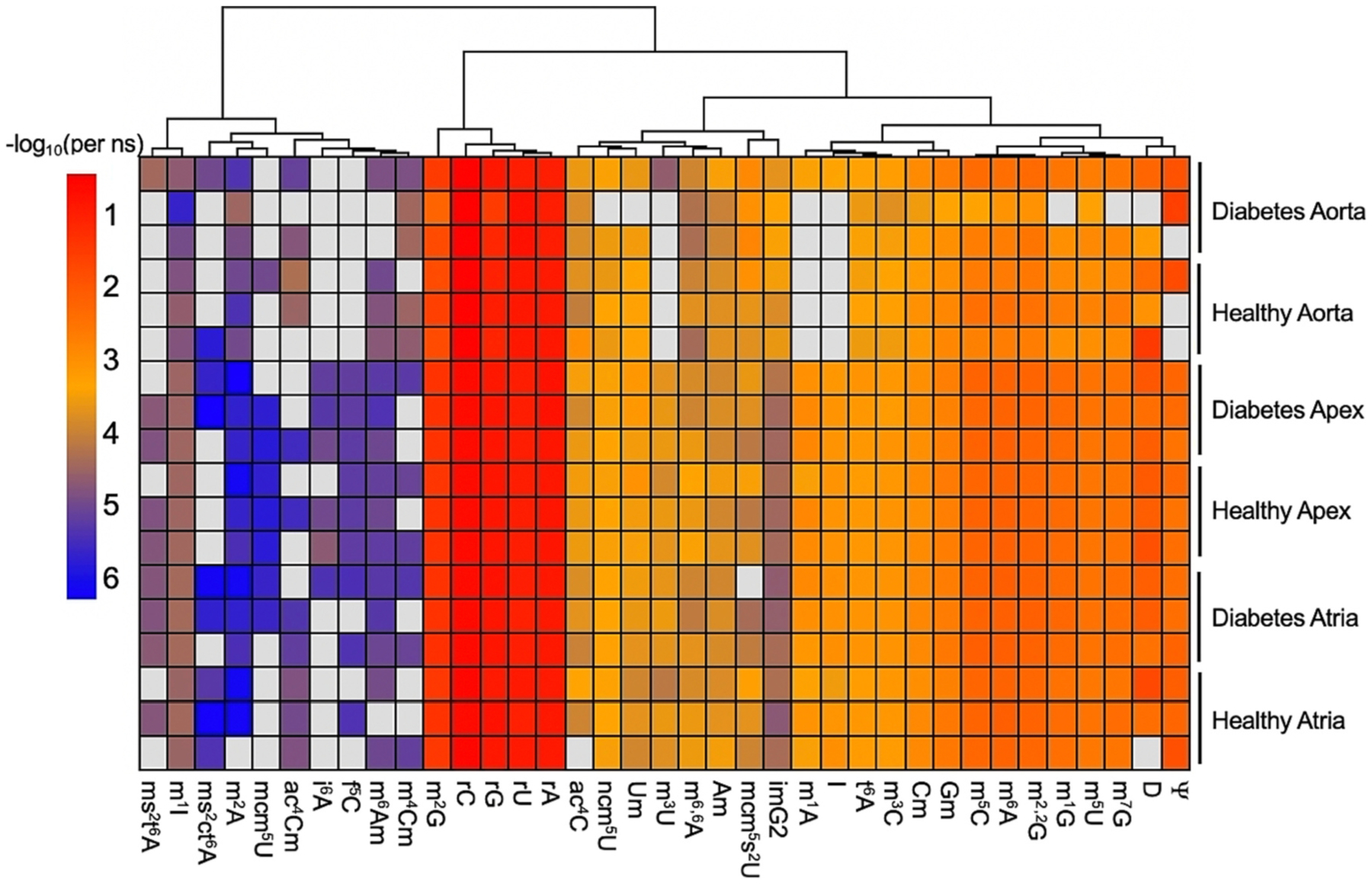
Hierarchical clustering of tRNA ribonucleoside levels (in –log_10_(per nucleoside)) for each biological replicate analyzed from healthy or diabetic murine heart tissue. Row clustering is set to false. Gray boxes indicate levels are below the LOD (Supplementary Table S3).

**Fig. 3.**
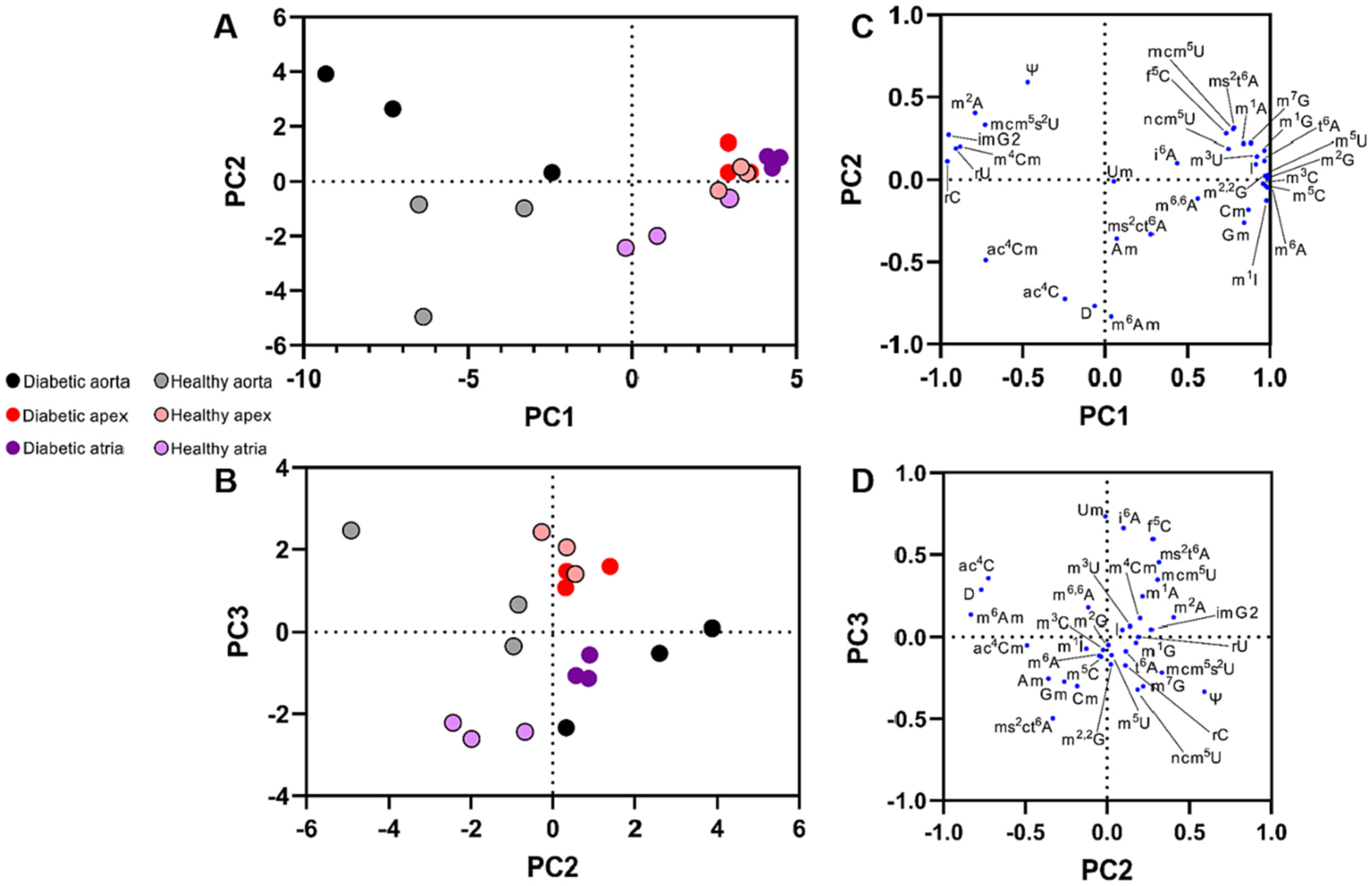
Principal component analysis (PCA) of tRNA ribonucleoside levels in diabetic and healthy murine heart tissue. Diabetes-induced changes in relative levels of 35 tRNA ribonucleosides were subjected to PCA after standardization of data. A–B) PC scores depicting murine tissue bioreplicates. C–D) Loadings depicting tRNA ribonucleoside levels. PC1 covers 61 % of proportion of variance. PC2 covers 11 % of proportion of variance. PC3 covers 8 % of proportion of variance.

Relationships between ribonucleosides from murine tissues were visualized with hierarchical clustering (Fig. 2) and principal components (PCs) (Fig. 3A and B) and loadings (Fig. 3C and D) analyses. By PCA, the total variance expressed in the first three principal components were 80 % (61 %, 11 %, and 8 %, respectively). Hierarchical clustering and PCA revealed correlations between murine tissues and tRNA ribonucleoside levels. For example, levels of mcm^5^s^2^U were higher in diabetic aorta than healthy aorta (Table 1). PCA revealed that in addition to mcm^5^s^2^U, diabetic aorta was correlated with levels of N^2^-methyladenosine (m^2^A), isowyosine (imG2), N^4^-methyl-2^′^-*O*-methylcytidine (m^4^Cm), rU, pseudouridine (Ψ), and rC (Fig. 3A, C). Modifications ncm^5^U, mcm^5^U, 5-formylcytidine (f^5^C), 2-methylthio-N^6^-threonylcarbamoyladenosine (ms^2^t^6^A), t^6^A, N^1^-methyladenosine (m^1^A), N^7^-methylguanosine (m^7^G), m^1^G, N^2^-methylguanosine (m^2^G), N^2^,N^2^,-dimethylguanosine (m^2,2^G), inosine (I), N^5^-methyluridine (m^5^U), and N^3^-methyluridine (m^3^U) were associated with diabetes in both murine apex and atria (Fig. 3A, C). Evidence indicates that tRNA modification-associated diabetes etiology is tissue specific.

We report m^6^Am, cyclic 2-methylthio-N^6^-threonylcarbamoyladenosine (ms^2^ct^6^A), m^4^Cm, N^4^-acetyl-2^′^-*O*-methylcytidine (ac^4^Cm), imG2 (isobar of i^6^A), and N^1^-methylinosine (m^1^I) as tRNA ribonucleosides in murine heart tissue (Supplementary Table S3) which have not yet been identified in mouse tissue tRNA (Supplementary Table S4) [9,10,23,41,42]. We used MS fragmentation to confirm the identities of these ribonucleoside modifications (Supplementary Figs. S5 and S8). We did not detect 2-methylthio-N^6^-isopentenyladenosine (ms^2^i^6^A) [23,43] though this MRM was included in our LC-MS/MS method. Here, m^1^I and m^6^Am were identified in aortic, apex, and atrial tissue tRNA (Fig. 2 and Supplementary Table S3). The ribonucleoside modifications m^1^I and m^6^Am have been previously identified in small RNA under 200 nucleotides in murine tissue, but not specifically in tRNA [44]. The ribonucleoside modifications m^4^Cm and imG2 were correlated with diabetic aorta (Fig. 3A and C). These newly reported tRNA nucleoside modifications were closely associated with specific murine heart tissues.

The modified ribonucleoside imG2 was discovered here in tRNA from aorta, atria, and apex (Fig. 2 and Supplementary Table S3) but previously has only been found in archaea [2]. In archaea, the enzyme Taw22/Trm5a has bifunctionality of catalyzing the biosynthesis of m^1^G and imG2 [45]. The N^1^-guanine-methyltransferase mammalian ortholog (Trm5) has roughly 31 % similar identity to archaeal Trm5a (BLAST). It is possible that mammalian Trm5 may have a similar bifunctionality in mammalian cells (Supplementary Fig. S9). It is also possible that we detected an unidentified ribonucleoside with the same MRM and retention time as imG2.

We identified m^4^Cm and ac^4^Cm in tRNA (Fig. 2, Supplementary Fig. S8, Supplementary Tables S2 and S3), though these were previously identified as ribosomal RNA (rRNA) modifications [2]. Some detected tRNA modifications such as Am, m^3^U, and m^6,6^A, were predicted to originate from degraded rRNA [23]. It is also possible that the modifications detected here for the first time in murine tRNA could also be caused by contamination of degraded RNA.

The ribonucleoside modification ms^2^ct^6^A (MRM *m*/*z* 441 > 309) was identified in murine tissue (Supplementary Fig. S5) and was correlated with healthy atria (Fig. 3). The oxazolone isoform of cyclic N^6^-threonylcarbamoyladenosine (ct^6^A) was previously reported to be endogenous to *Escherichia coli* but was identified as the hydantoin isoform [46]. The hydantoin isoform of ms^2^ct^6^A has been confirmed in *Bacillus subtilis* and *Trypanosoma brucei* tRNA^Lys(UUU)^ with characteristic collision induced dissociation (CID) fragment ions *m*/*z* 182, 208, 265, and 281 [47]. Here in murine tissue, we see fragment ions *m*/*z* 120, 129, 142, 187, and 276 (Supplementary Fig. S5) from a product ion scan of *m*/*z* 441 (putative isoxazoline isoform of ms^2^ct^6^A, MRM *m*/*z* 441 > 309).

Some ribonucleoside modifications which increased in diabetic heart tissue were closely clustered (Fig. 2). For example, modifications m^5^C, m^1^G, t^6^A, m^1^A, m^2^G, and m^7^G were closely clustered (Figs. 1B and 2), correlated with diabetic atrial tissue via PCA (Fig. 3), and levels were higher in diabetic atria than healthy atria (Table 1, Figs. 1B and 2). There is one murine tRNA known to contain m^7^G, m^1^G, t^6^A, m^1^A, m^2^G, and m^5^C: tRNA^Ini(CAU)^ [2]. Murine tRNA^Ini^ was previously identified as a substrate for enzymes tRNA methyltransferase 10A (Trmt10A), which catalyzes the biosynthesis of m^1^G (Supplementary Fig. S10), and AlkBH1, which preferentially demethylates m^1^A in tRNA [48].

## 4. Discussion

This study evaluated tRNA ribonucleosides in a type II diabetes mouse model. The established type II diabetic model is characterized by pancreatic β-cell failure, impaired glucose regulation, obesity, and hyperlipidemia [18]. Cardiac tissues were analyzed at initial changes in cardiac function and glucose intolerance, consistent with the earliest stages of cardiomyopathy. Gender differences were not evaluated in this study; however, streptozotocin has been shown to similarly impact peripheral tissues like retinopathy regardless of gender [49]. The differences observed in modified and canonical ribonucleosides occur independent of observable pathology.

Deficiency of Trmt10A (and subsequent reduction of m^1^G), induces oxidative stress and triggers apoptosis in pancreatic β-cells [50]. Additionally, Trmt10A deficiency contributes to impaired glucose regulation [51] and the pathogenesis of early onset diabetes by negatively impacting β-cell mass [52]. Here, the opposite trend is reported as higher murine atrial m^1^G levels result from diabetes (Table 1). This suggests that Trmt10A activity in diabetes may be tissue specific.

Glucose deprivation induces AlkBH1 expression in HeLa cells, leading to lower tRNA levels of m^1^A [48]. Our work further demonstrates this correlation between glucose and m^1^A levels in apex and atria (Fig. 3A and C). Expression levels of tRNA^Ini^ also decreased with glucose deprivation in HeLa cells, causing translational repression [48]. This may indicate higher expression of tRNA^Ini^ and subsequent induction of translation in murine diabetic atrial tissue than in healthy atrial tissue.

Some modified ribonucleosides were below the LOD in some cell or tissue types. For example, m^1^A was below the LOD in MEK cells and aortic tissue (Supplementary Tables S2 and S3). We hypothesized that one possible cause of low m^1^A levels could be due to pH-dependent Dimroth rearrangement of m^1^A to m^6^A [53]. Controls testing our extraction process determined m^1^A and m^6^A levels did not significantly change. This suggests that m^1^A differences were not due to Dimroth rearrangement (Supplementary Fig. S11). Likely, differences in tRNA modification profiles between samples were caused by mechanisms that are tissue or cell specific.

Hierarchical clustering and PCA revealed other tRNA modification tissue-specific trends. The modified ribonucleosides Cm and Gm were closely clustered (Figs. 1B and 2) and were correlated with healthy atrial tissue (Fig. 3). Both Cm and Gm have been identified on five murine tRNA species (three tRNA^Phe^ isodecoders, and two tRNA^Gln^ isoacceptors) [2]. The enzyme, Ftsj1, is responsible for catalyzing the 2^′^-*O*-methylation of cytidine and guanosine. It has been suggested that Ftsj1 activity relies on nucleoporin Nup155 [54]. However, mutations to nucleoporin Nup155 are associated with atrial fibrillation [55]. This implies that normal nucleoporin Nup155 and Ftsj1 activity are necessary for healthy atrial function. The correlations seen here between healthy murine atria and tRNA modifications Cm and Gm, further suggest that Ftsj1 is necessary for proper atrial function in mice.

The enzyme, NAT10, is responsible for the incorporation of an acetyl moiety on the fourth position of cytidine (Supplementary Fig. S10) and has been identified as a potential target for the treatment of diseases such as cancer [56], Hutchinson-Gilford Progeria Syndrome [57], and osteoporosis [41]. It was previously reported that hydrogen peroxide (H_2_O_2_) treatment of human cell lines induced NAT10 by activation of the NAT10 gene promoter, indicating DNA damage due to genotoxicity [12]. Levels of ac^4^C were higher in all cell lines cultured in high glucose media (Fig. 1B and Supplementary Table S2), though ac^4^C levels were lower in all diabetic heart tissue (Fig. 1B and Supplementary Table S3). This indicates that NAT10 expression or activity of *in vitro* mammalian cells and heart tissue could be regulated by different mechanisms or differentially influenced by ROS.

The pathways involved in the formation of uridine modifications, mcm^5^s^2^U and ncm^5^U, are interconnected (Supplementary Fig. S10). Uridine wobble modifications such as thiolations, methoxycarbonylmethylations, 5-carbomoylmethylations, or 5-methoxycarbonyl-methyl-2-thiolations [58–60] generally confer eukaryotic cell protection against oxidative stress [61,62]. The tRNA methyltransferase, Alkbh8, is necessary for the biosynthesis of mcm^5^s^2^U (Supplementary Fig. S10), however, the *Alkbh8−*/− phenotype corresponds with increased levels of ncm^5^U [10]. The *Alkbh8*−/− phenotype is associated with higher levels of ROS in mouse embryonic fibroblasts [9]. Thus, in mouse embryonic fibroblasts, higher levels of ncm^5^U may confer cell protection against ROS. Here, mcm^5^s^2^U levels were significantly higher in diabetic aorta than in healthy aorta (Table 1). Similarly, mcm^5^s^2^U was correlated with diabetic aorta via PCA (Fig. 3A and C). This suggests that uridine modifications such as mcm^5^s^2^U or ncm^5^U are potentially important for cellular protection against ROS. A higher level of mcm^5^s^2^U in diabetic aorta could be a mechanistic response to increased oxidative stress caused by hyperglycemia.

The eukaryotic enzymes Dnmt2, NSun2 [63], and Trm4 [64] incorporate a methyl moiety onto the fifth position of cytidine to biosynthesize m^5^C (Supplementary Fig. S10). In human fibroblasts, Dnmt2 knockdowns were susceptible to H_2_O_2_ and demonstrated increased protein carbonylation [65]. In other work, yeast exposed to H_2_O_2_ showed increased levels of m^5^C in total tRNA, which in turn enhanced translation of proteins. Among the yeast proteins affected by H_2_O_2_ exposure, stress response and translational proteins were significantly upregulated [11]. This reasonably suggests that the significantly higher levels of m^5^C in diabetic atrial tissue (Table 1) could be a mechanistic response to increased ROS formation. Similarly, this indicates that oxidative stress caused by hyperglycemia may influence protein modification and translation in murine atria. Addressing the causal role of tRNA modifications in chronic diabetes models over long periods will inform our understanding of metabolic regulation of ribonucleoside modifications and implications for disease progression.

## 5. Conclusions

It is important to identify tRNA ribonucleoside modifications associated with disease pathogenesis [4]. Oxidative stress caused by hyperglycemia can affect biomolecules associated with the incorporation of enzymatic chemical modified ribonucleosides. We identified and quantified tRNA ribonucleosides from *in vitro* and *in vivo* models to analyze the consequences of hyperglycemia. It is also of future interest to identify tRNA expression levels and tRNA modifications specific to tRNA species, especially tRNA^Ini^, to identify downstream translational effects of hyperglycemia [48]. Statistical analyses demonstrated tRNA modification profiles associated with healthy and diabetic cardiac murine tissues. We discuss enzymes likely affected by hyperglycemia, as results indicate ribonucleosides, ac^4^C, mcm^5^s^2^U, m^5^C, and m^1^G were aberrant in hyperglycemic models. We characterized tRNA species, tRNA ribonucleoside modifications, and potential tRNA modifying enzymes associated with diabetic etiology.

## Supplementary Material

Supplement

## Figures and Tables

**Schematic 4.**
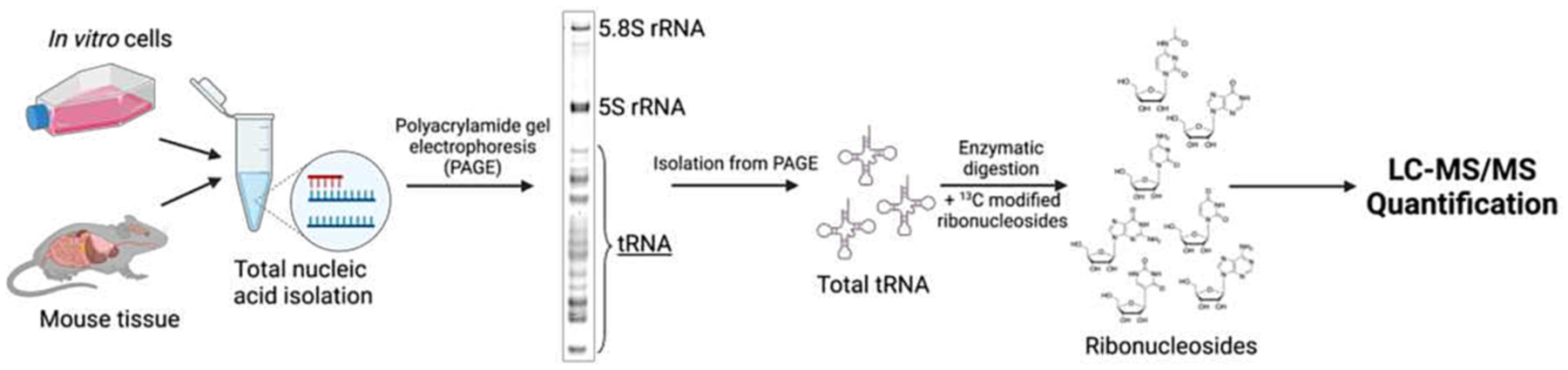
Isolation of total tRNA from *in vitro* cells and murine tissue for LC-MS/MS quantification.

**Table 1:** Ribonucleoside levels found to be significantly different in mammalian *in vitro* cells grown in low or high glucose media, and diabetic or control murine tissue via LC-MS/MS quantification. A Student’s unpaired *t*-test was performed to determine significance.

*In vitro* cells					Murine tissue				
Cell type	Ribonucleoside	*P* value	Mean of high glucose (per 10^4^ ns)	Mean of low glucose (per 10^4^ ns)	Tissue	Ribonucleoside	*P* value	Mean of diabetes (per 10^4^ ns)	Mean of control (per 10^4^ ns)
3T3	ac^4^C	0.008	490.0 ± 8.34	452.2 ± 10.49	Aorta	Um	0.022	3.49 ± 0.48**	6.17 ± 0.43
	Adenosine	0.01	1665.0 ± 26.1	1595.4 ± 27.4		mcm^5^s^2^U	0.034	17.11 ± 3.49	6.02 ± 4.71
	m^6^Am	0.022	0.075 ± 0.010	0.048 ± 0.0075		Adenosine	0.041	1430.58 ± 15.42	1744.92 ± 116.23
	mcm^5^U	0.049	0.061 ± 0.010	0.082 ± 0.0075	Apex	m^6,6^A	0.031	2.24 ± 0.81	4.60 ± 0.94
H9C2	t^6^A	0.080*	12.15 ± 1.83	7.24 ± 4.57	Atria	m^3^C	0.0056	11.16 ± 0.28	9.39 ± 0.42
	D	0.066*	167.4 ± 21.3	136.3 ± 17.3		m^5^C	0.0077	91.09 ± 4.07	73.91 ± 4.38
	Guanosine	0.054*	2374.0 ± 602.0	3095.6 ± 233.9		ac^4^Cm	0.0095	0.071 ± 0.014**	0.17 ± 0.025
MEK	m^3^U	0.018	4.65 ± 0.682	3.25 ± 0.344		t^6^A	0.013	10.87 ± 0.28	8.70 ± 0.60
	Am	0.0015	12.8 ± 1.19	4.67 ± 0.685		Um	0.032	3.170 ± 0.44	1.95 ± 0.48
	m^2,2^G	0.0096	51.0 ± 5.68	67.8 ± 4.95		m^1^G	0.038	65.17 ± 1.23	51.09 ± 5.26
	m^2^G	0.0035	304.6 ± 55.9	482.3 ± 29.5		m^6^Am	0.049	0.082 ± 0.024	0.14 ± 0.014**
	m^5^C	0.0094	54.0 ± 5.87	78.7 ± 9.81					
	m^1^G	0.013	45.1 ± 5.39	67.8 ± 7.23					
	Adenosine	0.017	1939.3 ± 11.7	1849.7 ± 26.4					
	t^6^A	0.018	7.86 ± 1.35	11.4 ± 1.28					

Significance is to 95 % confidence interval, except 90 % confidence interval*.

Data presented as the mean and std. deviation of at least 3 biological replicates, except when denoted**, then 2 biological replicates.
