# Supplementary material for: Ribonucleosides from tRNA in hyperglycemic mammalian cells and diabetic murine cardiac models
Source: Life Sci. Author manuscript; Available in PMC 2024 Apr 1. (PMC9992345; doi:10.1016/j.lfs.2023.121462)
Supplement: Supplement [file NIHMS1876565-supplement-Supplement.pdf]

Supporting Information

Ribonucleosides from tRNA in Hyperglycemic Mammalian Cells and Diabetic Murine Cardiac Models

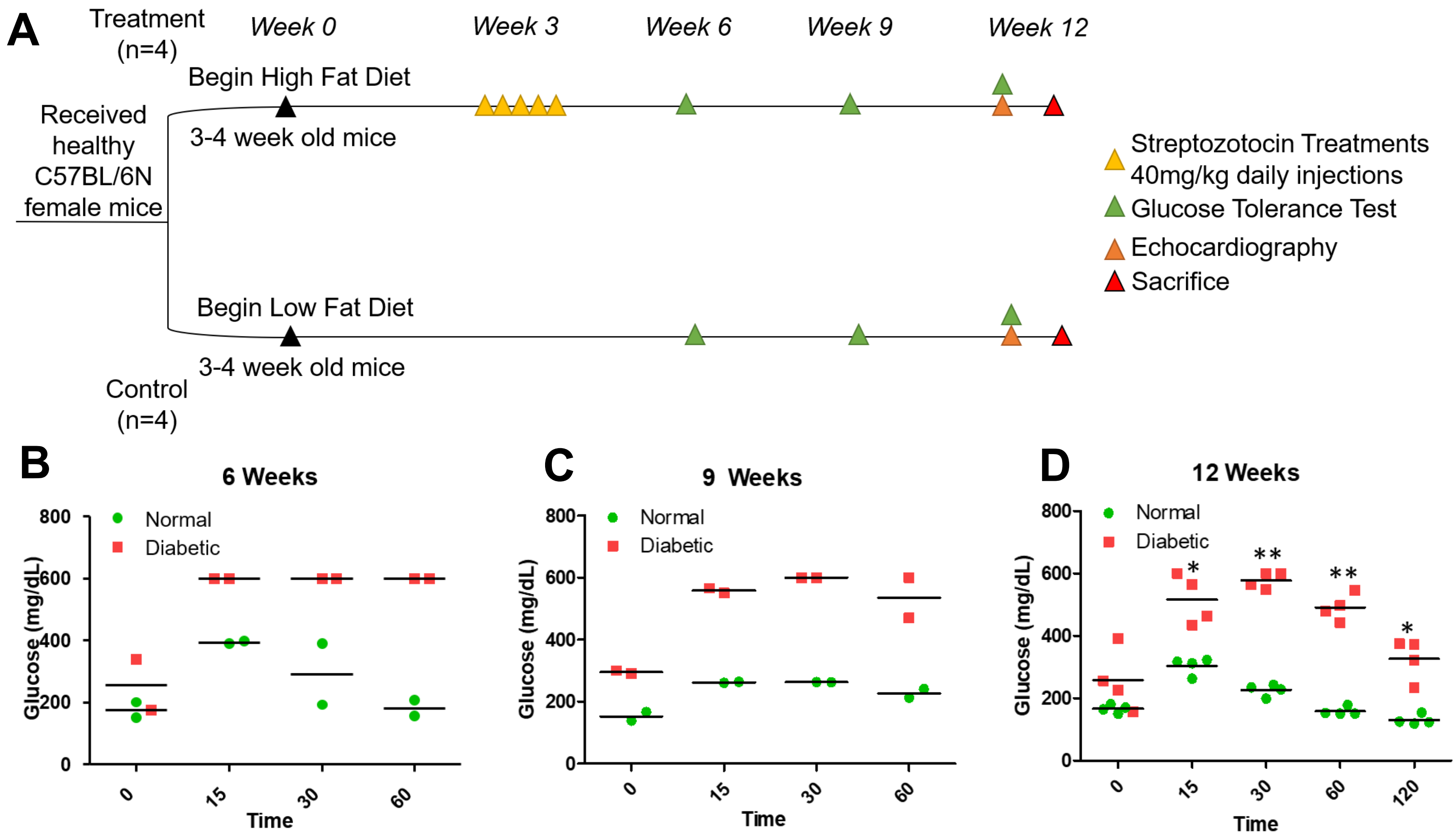

Supplementary Figure S1: Tail vein glucose tolerance test. All mice were evaluated after the diabetes mouse model received streptozotocin injection to induce pancreatic β-cell failure followed by conventional high fat diet. Glucose tolerance injection of dextrose (0.25 mL max volume) at 2 mg/g into fasting mice with blood samples collected at 0, 15, 30, 60, and 120 mins from the tail vein (repeated sampling after snip) for blood glucose measurement. Glucose levels were confirmed with the glucose tolerance test at weeks 6, 9, and 12 after 6-hour fasts. Glucose regulation is impaired as observed at 6 weeks and beyond. Multiple t-test per time points for week 12 (*$p<0.005$; **$p<0.0001$).

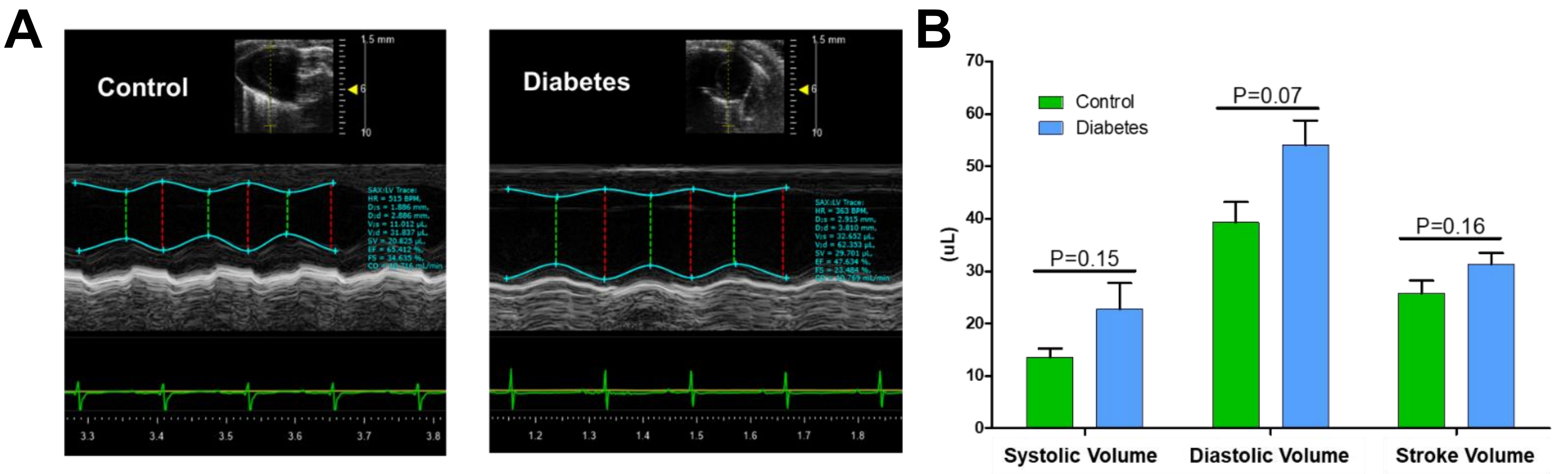

Supplementary Figure S2: Cardiac function was evaluated in all mice by echocardiography under anesthesia at 12 weeks. Diabetic mice exhibited trends towards reduced ejection fraction and impaired diastole indicative of early stages of cardiomyopathy. Graphical representation of volume measurements. Comparing healthy diet mice to the diabetes model we observe changes in systolic volume (13.56 ± 2.86 vs 22.75 ± 8.58), diastolic volume (39.31 ± 6.63 vs 54.08 ± 8.05) and stroke volume (25.74 ± 4.28 vs 31.34 ± 3.69) with trends for impairment in diastolic function. No statistical significance was observed using an unpaired two tailed t-test, (n = 3).

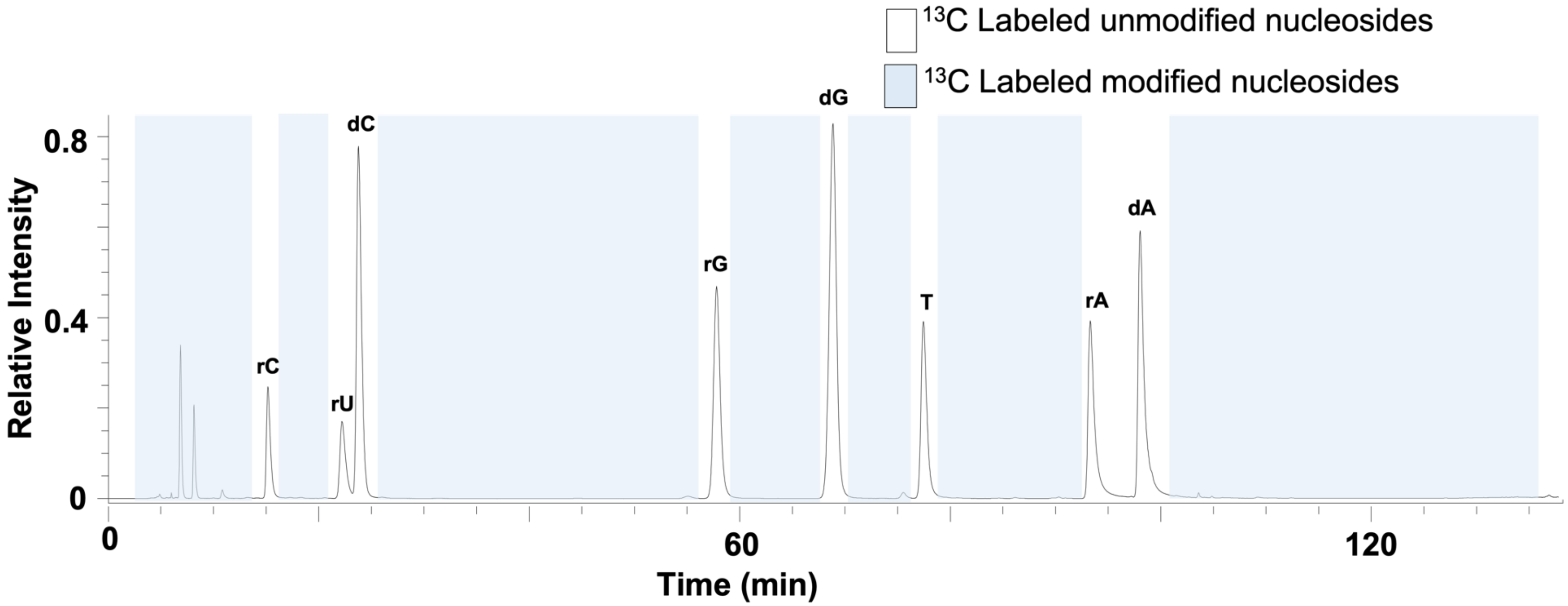

Supplementary Figure S3: HPLC chromatographic separation of ^{13}C labeled nucleosides. Blue indicates the retention of modified labeled nucleosides which were fractionated and combined. White indicates the retention of unmodified canonical labeled nucleosides which were fractionated separately from the modified labeled nucleosides (blue). This method allowed for the biosynthesis of 21 isotopically labeled modified ribonucleosides, including N^6,N^6-dimethyladenosine ($m^{6,6}A$), N^6-threonylcarbamoyladenosine (t^6A), C^2-methyladenosine (m^2A), 2'-O-methyladenosine (Am), N^6-methyladenosine (m^6A), N^6-(3-methyl-2-butenyl)adenosine (i^6A), 5-methylcytidine (m^5C), 2-thiocytidine (s^2C), 2'-O-methylcytidine (Cm), N^4-methyl-2'-O-methylcytidine (m^4Cm), dihydrouridine (D), pseudouridine (Ψ), 5-methyluridine (m^5U), 4-thiouridine (s^4U), N^3-methyluridine (m^3U), 2'-O-methyluridine (Um), 7-methylguanosine (m^7G), N^2-methylguanosine (m^2G), N^1-methylguanosine (m^1G), 2'-O-methylguanosine (Gm), and inosine (I).

**Supplementary Table S1:** All modified ribonucleosides monitored by LC-MS/MS with nomenclature, MRM, LOD, and LOQ.						
**Nucleoside**	**Shorthand**	**MRM**	**LOD (fmol)**	**LOQ (fmol)**	**Structure**	**Purchased from**
N^6,N^6-dimethyladenosine	$m^{6,6}A$	**296>164**	0.49 ± 0.23	0.54 ± 0.20	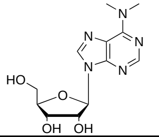	Toronto Research Chemicals
N^6-2'-O-dimethyladenosine	m^6Am	**296>150**	0.40 ± 0.10 (fmol)<br>Diabetes aorta 0.41 x 10^{-4} ± 0.14 x 10^{-4} per ns	0.50 ± 0	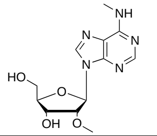	Toronto Research Chemicals
N^1-2'-O-dimethyladenosine	m^1Am	**296>150**	0.30 ± 0	0.30 ± 0	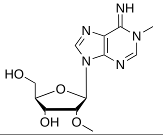	Toronto Research Chemicals
N^6,N^6-2'-O-trimethyladenosine	$m^{6,6}Am$	**310>164**	0.30 ± 0	0.67 ± 0.24	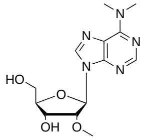	Carbosynth
N^6-(N-threonylcarbonyl) adenosine	t^6A	**413>281**	0.46 ± 0.08	1.6 ± 1.71	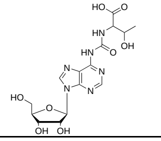	Toronto Research Chemicals
2-methylthio-N^6-isopentenyl-adenosine	ms^2i^6A	**382>250**	0.83 ± 0.24	5.0 ± 0	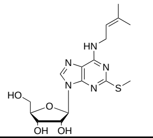	Carbosynth
2-methylthio-N^6-methyladenosine	ms^2m^6A	**328>196**	0.30 ± 0	0.83 ± 0.24	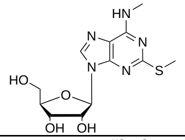	Carbosynth
N^6-methyl-N^6-threonylcarbamoyl adenosine	m^6t^6A	**427>295**	No standard	No standard	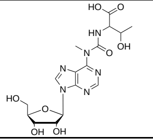	NA

2-methylthio-N^6-threonylcarbamoyl adenosine	ms^{2}t^{6}A	**459>327**	No standard	No standard	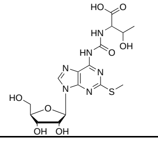	NA
N^6-(cis-hydroxyisopentenyl) adenosine	io^{6}A	**352>220**	No standard	No standard	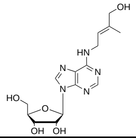	NA
2-methylthio-N^6-(cis-hydroxyisopentenyl) adenosine	ms^{2}io^{6}A	**398>266**	No standard	No standard	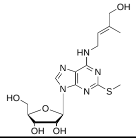	NA
Cyclic 2-methylthio-N^6-threonylcarbamoyl adenosine	ms^{2}ct^{6}A	**441>309**	No standard	No standard	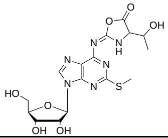	NA
Cyclic N^6-threonylcarbamoyl adenosine	ct^{6}A	**395>263**	No standard	No standard	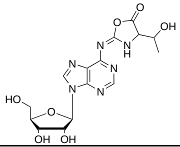	NA
N^1-methyladenosine	m^{1}A	**282>150**	0.50 ± 0 (fmol)<br>MEK LG 0.12 x 10^{-4} ± 0.13 x 10^{-4} per ns<br>MEK HG 0.066 x 10^{-4} ± 0.036 x 10^{-4} per ns<br>Diabetes aorta 0.51 x 10^{-4} ± 0.17 x 10^{-4} per ns<br>Control aorta 0.50 x10^{-4} ± 0.032 x 10^{-4} per ns	1.0 ± 0	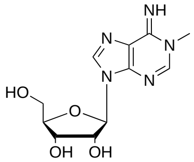	Carbosynth

C^2-methyladenosine	m^2A	**282>150**	0.41 ± 0.1 (fmol)<br>MEK LG 0.10 x 10^{-4} ± 0.11 x 10^{-4} per ns<br>MEK HG 0.054 x 10^{-4} ± 0.030 x 10^{-4} per ns	0.50 ± 0	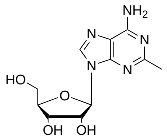	Carbosynth
2'-O-methyladenosine	Am	**282>136**	0.36 ± 0.18	0.41 ± 0.146	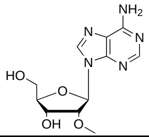	Carbosynth
N^6-methyladenosine	m^6A	**282>150**	0.54 ± 0.2	0.57 ± 0.17	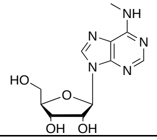	Carbosynth
C^8-methyl-adenosine	m^8A	**282>150**	0.30 ± 0	3.5 ± 2.12	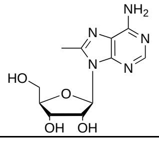	Carbosynth
N^6-(3-methyl-2-butenyl)adenosine	i^6A	**336>204**	0.44 ± 0.09 (fmol)<br>Diabetes aorta 0.45 x 10^{-4} ± 0.15 x 10^{-4} per ns<br>Control aorta 0.44 x 10^{-4} ± 0.028 x 10^{-4} per ns<br>Diabetes atria 0.10x10^{-4} ± 0.017x10^{-4}per ns<br>Control atria 0.17 x 10^{-4} ± 0.049 x 10^{-4} per ns	0.79 ± 0.25	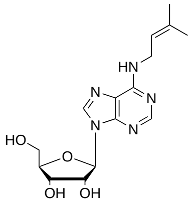	Ark Pharm
N^6-formyladenosine	f^6A	**296>164**	0.30 ± 0	0.30 ± 0	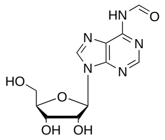	Toronto Research Chemicals

5-formylcytidine	f^5C	**272>140**	0.30 ± 0 (fmol)<br>Diabetes aorta 0.31 x 10^{-4} ± 0.10 x 10^{-4} per ns<br>Control aorta 0.30 x10^{-4} ± 0.020 x 10^{-4} per ns<br>Control atria 0.11 x 10^{-4} ± 0.033 x 10^{-4} per ns	0.30 ± 0	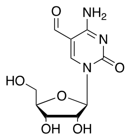	Carbosynth
5-formyl-2'-O-cytidine	f^5Cm	**286>140**	0.75 ± 0.25 (fmol)<br>MEK LG 0.19 x 10^{-4} ± 0.20 x 10^{-4} per ns<br>MEK HG 0.10 x 10^{-4} ± 0.054 x 10^{-4} per ns	0.75 ± 0.25	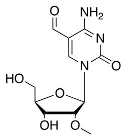	Carbosynth
5-methylcytidine	m^5C	**258>126**	0.36 ± 0.178	0.66 ± 0.31	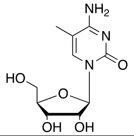	Carbosynth
N^4-methylcytidine	m^4C	**258>126**	0.63 ± 0.28	0.73 ± 0.27	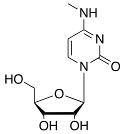	Carbosynth
N^3-methylcytidine	m^3C	**258>126**	0.37 ± 0.09	0.67 ± 0.24	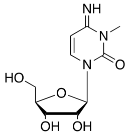	Carbosynth
2-thiocytidine	s^2C	**260>128**	2.66 ± 2.99	2.73 ± 2.94	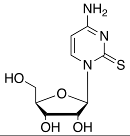	Carbosynth
2'-O-cytidine	Cm	**258>112**	0.41 ± 0.1	0.54 ± 0.2	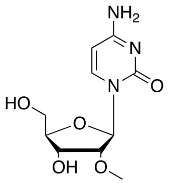	Carbosynth

N^{4}-methyl-2’-O-cytidine	m^{4}Cm	**272>126**	0.54 ± 0.2 (fmol)<br>3T3 HG 0.038 x 10^{-4} ± 0.014 x 10^{-4} per ns<br>3T3 LG 0.065 x 10^{-4} ± 0.013 x 10^{-4} per ns<br>MEK LG 0.13 x 10^{-4} ± 0.14 x 10^{-4} per ns<br>MEK HG 0.071 x 10^{-4} ± 0.039 x 10^{-4} per ns<br>Diabetes apex 0.13 x 10^{-4} ± 0.031x10^{-4}per ns<br>Control atria 0.21 x 10^{-4} ± 0.050 x 10^{-4} per ns	0.86 ± 0.23	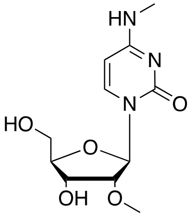	Carbosynth
N^{5}-methyl-2’-O-cytidine	m^{5}Cm	**272>126**	Not monitored during quantitative analysis	Not monitored during quantitative analysis	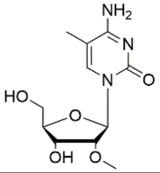	Toronto Research Chemicals
N^{4}-acetylcytidine	ac^{4}C	**286>154**	0.30 ± 0	0.30 ± 0	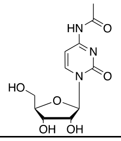	Ark Pharm
N^{4}-acetyl-2’-O-methylcytidine	ac^{4}Cm	**300>154**	0.30 ± 0	0.30 ± 0	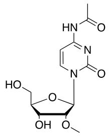	Sigma-Aldrich
Dihydrouridine	D	**247>115**	3.23 ± 5.05	5.87 ± 4.53	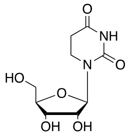	Carbosynth

5-methoxycarbonyl methyl-2-thiouridine	mcm^{5}s^{2}U	**333>201**	1.0 ± 0	1.0 ± 0	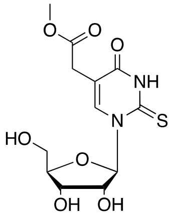	Carbosynth
5-methyl-2-thiouridine	m^{5}s^{2}U	**275>143**	1.0 ± 0 (fmol)<br>3T3 HG 0.071 x 10^{-4} ± 0.026 x 10^{-4} per ns<br>3T3 LG 0.12 x 10^{-4} ± 0.024 x 10^{-4} per ns<br>H9C2 HG 0.10 x 10^{-4} ± 0.011 x 10^{-4} per ns<br>H9C2 LG 0.15 x 10^{-4} ± 0.088 x 10^{-4} per ns<br>MEK LG 0.25 x 10^{-4} ± 0.27 x 10^{-4} per ns<br>MEK HG 0.13 x 10^{-4} ± 0.073 x 10^{-4} per ns	1.0 ± 0	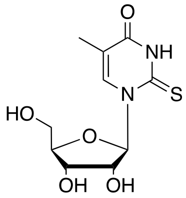	Carbosynth
5-methoxycarbonyl methyluridine	mcm^{5}U	**317>185**	0.45 ± 0.09 (fmol)<br>MEK LG 0.11 x 10^{-4} ± 0.12 x 10^{-4} per ns<br>MEK HG 0.059 x 10^{-4} ± 0.032 x 10^{-4} per ns<br>Diabetes aorta 0.46 x 10^{-4} ± 0.16x10^{-4} per ns<br>Control aorta 0.45 x 10^{-4} ± 0.029 x 10^{-4} per ns<br>Control atria 0.17 x 10^{-4} ± 0.050 x 10^{-4} per ns	0.50 ± 0	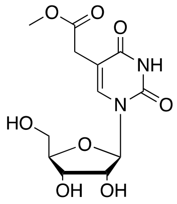	Carbosynth

5-carbamoyl methyluridine	ncm^{5}U	**302>170**	2.0 ± 1.73	2.0 ± 1.73	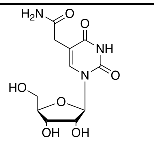	Carbosynth
5-hydroxyuridine	ho^{5}U	**261>129**	0.83 ± 0.24 (fmol)	5.0 ± 0	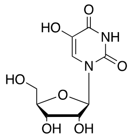	Carbosynth
Pseudouridine	Ψ	**245>209**	9.25 ± 5.19 (fmol)<br>3T3 HG 0.69 x 10^{-4} ± 0.25 x 10^{-4} per ns<br>3T3 LG 1.18 x 10^{-4} ± 0.23 x 10^{-4} per ns<br>H9C2 HG 0.98 x 10^{-4} ± 0.10 x 10^{-4} per ns<br>H9C2 LG 1.46 x 10^{-4} ± 0.86 x 10^{-4} per ns<br>MEK LG 2.41 x 10^{-4} ± 2.59 x 10^{-4} per ns<br>MEK HG 1.28 x 10^{-4} ± 0.71 x 10^{-4} per ns<br>Control aorta 9.71 x 10^{-4} ± 0.62 x 10^{-4} per ns	157.5 ± 142.5	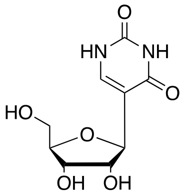	Carbosynth
3-methylpseudouridine	m^{3}Ψ	**259>223**	10.0 ± 0 (fmol)	25.0 ± 0	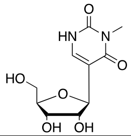	Carbosynth
1-methylpseudouridine	m^{1}Ψ	**259>223**	21.67 ± 4.71 (fmol)	25.0 ± 0	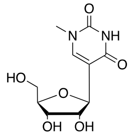	Carbosynth

5-methyluridine	m^5U	**259>127**	0.54 ± 0.20 (fmol)	0.73 ± 0.27	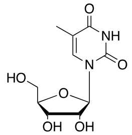	Ark Pharm
Uridine-5-oxyacetic acid	cmo^5U	**319>187**	5.5 ± 4.5 (fmol)	10.0 ± 0	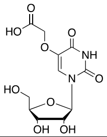	Carbosynth
2-thiouridine	s^2U	**261>129**	50.5 ± 49.5 (fmol)	54.55 ± 45.46	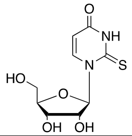	Carbosynth
4-thiouridine	s^4U	**261>129**	8.85 ± 5.8 (fmol)	8.85 ± 5.85	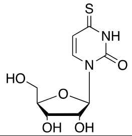	Carbosynth
5-methoxyuridine	mo^5U	**275>143**	15.0 ± 10.0 (fmol)	17.5 ± 7.5	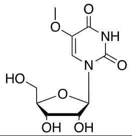	Carbosynth
N^3-methyluridine	m^3U	**259>127**	1.21 ± 1.56 (fmol)<br>Diabetes aorta 1.24 x10^{-4} ± 0.42x10^{-4} per ns<br>Control aorta 1.21 x10^{-4} ± 0.078 x 10^{-4} per ns	2.66 ± 2.99	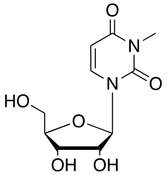	Carbosynth
2'-O-methyluridine	Um	**259>113**	2.06 ± 3.70 (fmol)	2.30 ± 3.60	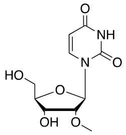	Sigma-Aldrich
3'-O-methyluridine	3'-O-methyluridine	**259>113**	0.60 ± 0.29 (fmol)	3.67 ± 1.89	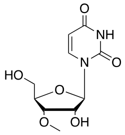	Sigma-Aldrich

N^2,N^7-dimethylguanosine	$m^{2,7}G$	**312>180**	0.30 ± 0 (fmol)	0.30 ± 0	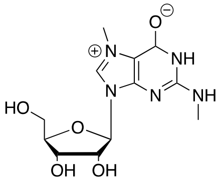	Carbosynth
Isowyosine	imG2	**336>204**	0.67 ± 0.24 (fmol)<br>3T3 HG 0.047 x 10^{-4} ± 0.017 x 10^{-4} per ns<br>3T3 LG 0.081 x 10^{-4} ± 0.016 x 10^{-4} per ns<br>H9C2 HG 0.066 x 10^{-4} ± 0.0070 x 10^{-4} per ns<br>MEK LG 0.17 x 10^{-4} ± 0.18 x 10^{-4} per ns<br>MEK HG 0.088 x 10^{-4} ± 0.049 x 10^{-4} per ns	0.83 ± 0.3	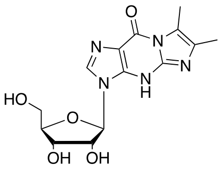	Toronto Research Chemicals
N^7-methylguanosine	m^7G	**298>166**	0.40 ± 0.15 (fmol)	0.43 ± 0.15	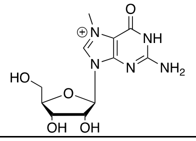	Sigma-Aldrich
N^2-methylguanosine	m^2G	**298>166**	0.54 ± 0.2 (fmol)	1.46 ± 1.47	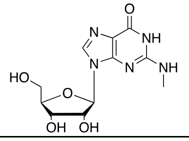	Carbosynth

N^1-methylguanosine	m^1G	**298>166**	0.41 ± 0.1 (fmol)	0.54 ± 0.2	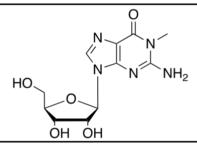	Carbosynth
2'-O-methylguanosine	Gm	**298>152**	0.37 ± 0.09 (fmol)	0.43 ± 0.09	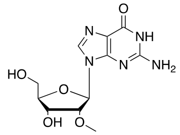	Carbosynth
N^2,N^2-dimethylguanosine	$m^{2,2}G$	**312>180**	0.30 ± 0 (fmol)	0.30 ± 0	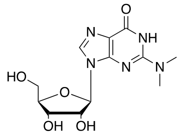	Ark Pharm
Inosine	I	**269>137**	0.30 ± 0 (fmol)<br>Diabetes aorta 0.31 x 10^{-4} ± 0.10 x 10^{-4} per ns<br>Control aorta 0.30 x10^{-4} ± 0.019 x 10^{-4} per ns	0.37 ± 0.09	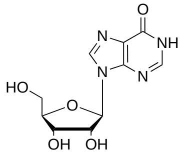	Carbosynth
N^1-methylinosine	m^1I	**283>151**	0.30 ± 0 (fmol)	0.30 ± 0	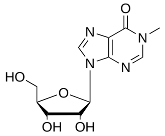	Carbosynth

2’-O-methylinosine	Im	**283>137**	0.30 ± 0 (fmol)<br>H9C2 HG 0.030 x 10^{-4} ± 0.0032 x 10^{-4} per ns<br>H9C2 LG 0.045 x 10^{-4} ± 0.026 x 10^{-4} per ns<br>MEK HG 0.039 x 10^{-4} ± 0.022 x 10^{-4} per ns	0.80 ± 0.24	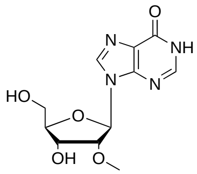	Carbosynth
Limit of detection (LOD) is the average of the lowest calibration point in regression curves (n ≥ 3) with a S/N ratio ≥ 3. LOD, per nucleosides (ns), were calculated by converting the LOD (in fmol) to the average ± std deviation of ns injected onto column per sample type. Limit of quantitation (LOQ) is the average of the lowest calibration point in regression curves (n ≥ 3) with a S/N ratio ≥ 10.						

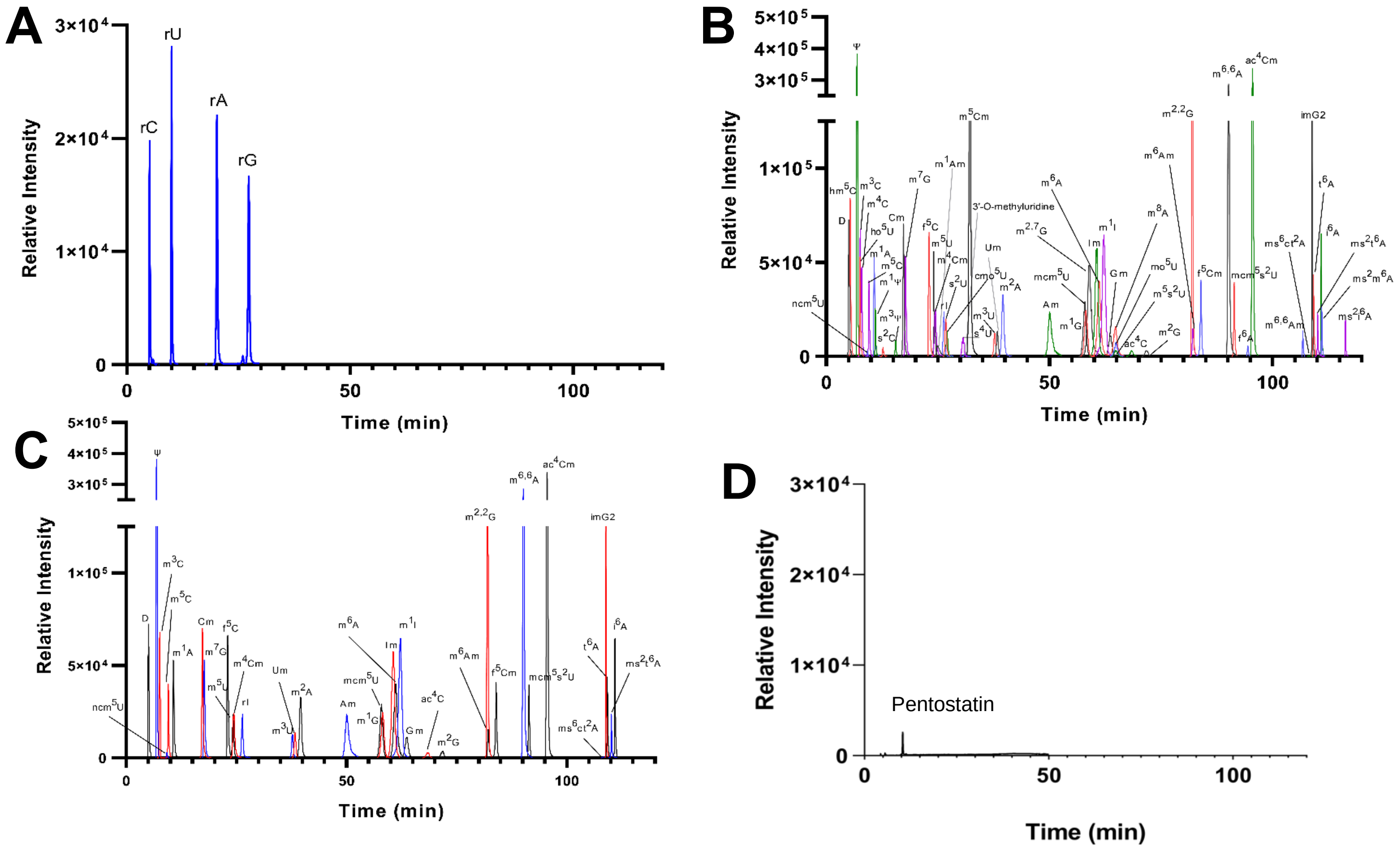

Supplementary Figure S4: HPLC chromatographic separation of ribonucleosides for simultaneous analysis by PDA and MS. Chromatograms represent the elution and separation of: A) Canonical ribonucleosides for quantitative PDA analysis B) 56 modified ribonucleosides for MS analysis C) Modified ribonucleosides detected in tRNA from *in vitro* MEK, 3T3, and H9C2 cells and/or murine heart tissue D) PDA analysis (260 nm) of ^{13}C isotopically labeled modified ribonucleosides, showing no UV detectable ^{13}C isotopically labeled canonical ribonucleosides.

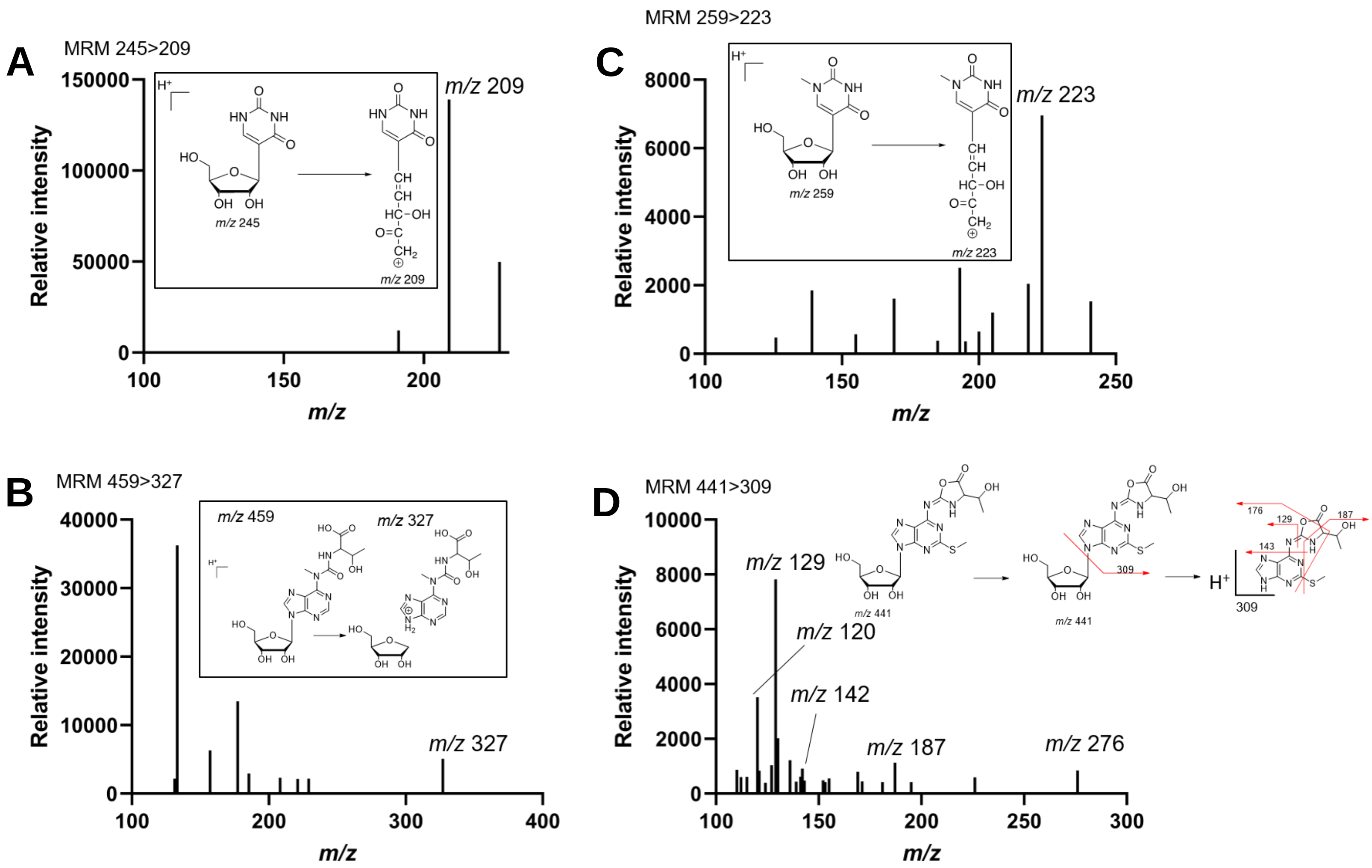

Supplementary Figure S5: Product ion scans depicting fragmentation patterns of A) pseudouridine (Ψ) (commercial standard), B) 2-methylthio-N^6-threonylcarbamoyl adenosine (ms^{2}t^{6}A) (endogenous), C) methyl-pseudouridine (commercial standard), and D) 2-methylthio cyclic -N^6-threonylcarbamoyladenosine (ms^{2}ct^{6}A) (endogenous).

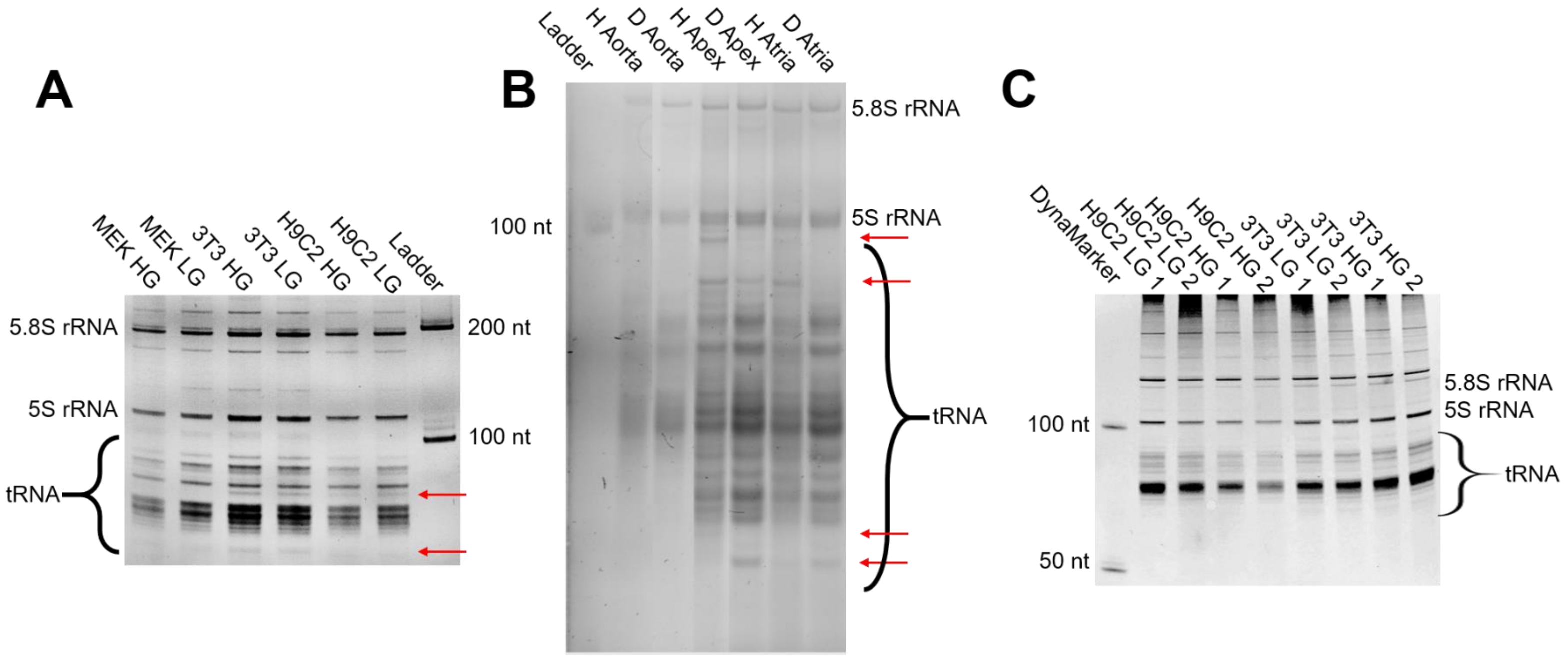

Supplementary Figure S6: A) Representative 15% urea denaturing PAGE separation of total isolated nucleic acid from MEK, 3T3, and H9C2 mammalian cells grown in high glucose (HG) or low glucose (LG) media. Gel contains RiboRuler Low Range RNA Ladder. B) Representative 20% urea denaturing PAGE separation of total isolated nucleic acid from aorta, apex, and atrial murine tissue from non-diabetic healthy (H) and diabetic (D) mice. Gel contains RiboRuler Low Range RNA Ladder. C) Representative 15% urea denaturing PAGE separation of total isolated nucleic acid from H9C2 and 3T3 mammalian cells ground in high glucose (HG) or low glucose (LG). Gel contains the DynaMarker, Small RNA II ladder. Red arrows depict visible tRNA band species abundance/presence differences between cell/tissue types.

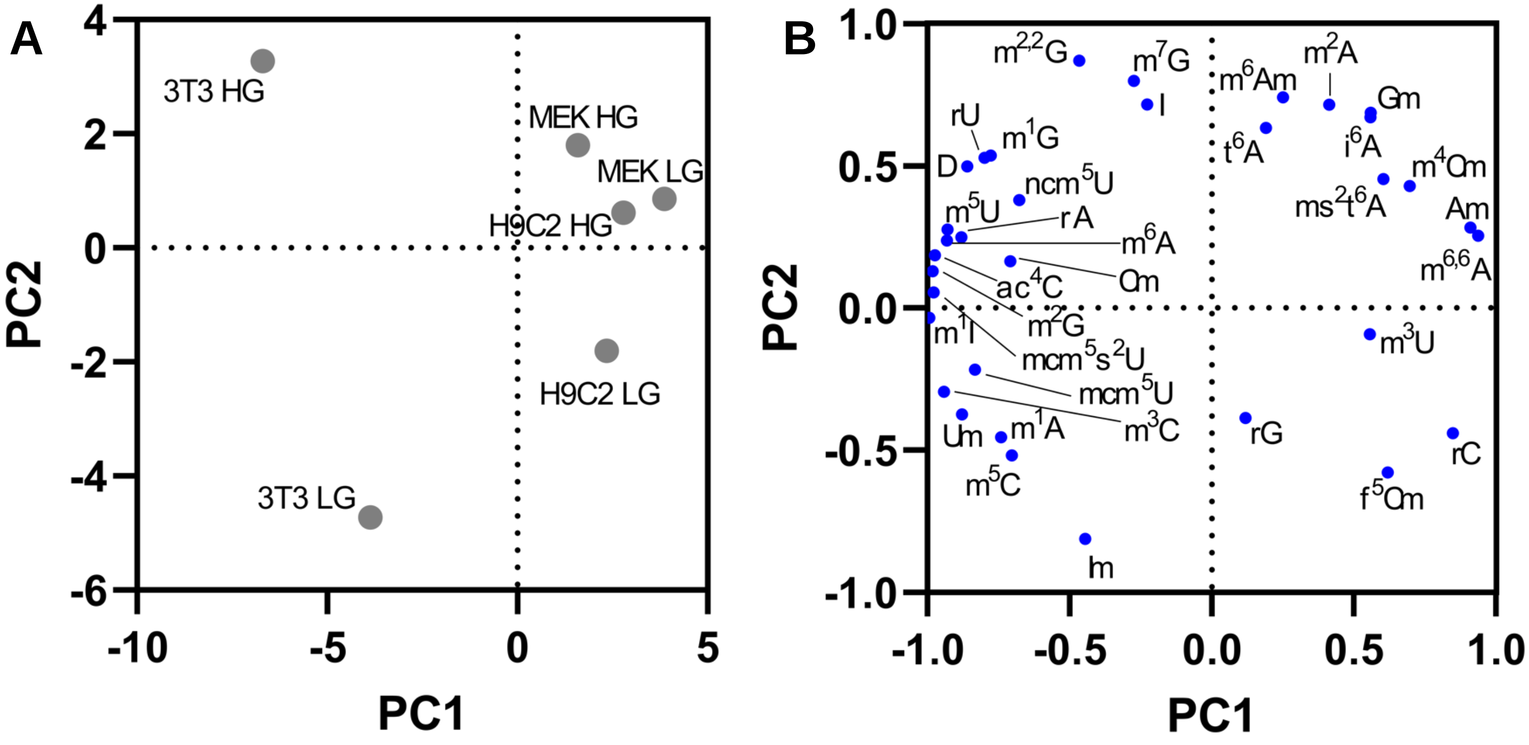

Supplementary Figure S7: Principal component analysis (PCA) of tRNA ribonucleoside levels in cells cultured in low or high glucose media. Glucose-induced changes in relative levels of 26 tRNA ribonucleosides were subjected to PCA after standardization of data. A) PC scores depicting *in vitro* cell averaged biological replicates. B) Loadings depicting tRNA ribonucleoside levels. PC1 covers 53% of proportion of variance. PC2 covers 24% of proportion of variance.

Supplementary Table S2: The average fmol per total injected nucleosides from *in vitro* cells of each ribonucleoside ± the standard deviation (in 10^4 ns). Count (N) represents biological replicates in which the nucleoside was > LOQ.

	**3T3 LG**			**3T3 HG**			**H9C2 LG**			**H9C2 HG**			**MEK LG**			**MEK HG**		
**Nucleoside**	**Average**	**Std Dev**	**N**	**Average**	**Std Dev**	**N**	**Average**	**Std Dev**	**N**	**Average**	**Std Dev**	**N**	**Average**	**Std Dev**	**N**	**Average**	**Std Dev**	**N**
rA	1595.38	27.41	4	1665.01	26.13	4	1559.72	241.05	5	1474.67	140.86	4	1849.74	26.39	3	1939.35	11.72	4
m^{6,6}A	0.40	0.0050	3	0.43	0.072	3	0.72	0.30	3	0.56	0.073	3	0.87	0.19	4	1.67	0.58	4
m^{6}Am	0.048	0.0010	3	0.075	0.010	3	0.074	0.026	3	0.046	0.013	3	0.12	0.017	3	0.17	0.049	4
t^{6}A	8.28	0.50	5	9.01	5.52	5	7.24	4.57	5	12.15	1.83	3	11.44	1.28	3	7.86	1.35	4
m^{1}A	7.80	0.034	3	7.71	1.03	3	7.62	0.52	3	7.85	0.71	3	Below LOD			Below LOD		
m^{2}A	0.025	0.001	3	0.147	0.177	3	0.011	0.005	3	0.005	0.005	3	Below LOD			Below LOD		
Am	2.72	0.11	4	2.74	0.75	4	3.47	2.29	4	2.70	1.33	4	4.67	0.69	3	12.83	1.19	3
m^{6}A	58.87	1.96	5	62.24	12.90	5	57.72	23.71	5	58.77	25.79	5	94.17	22.02	4	70.88	9.68	4
i^{6}A	0.50	0.010	4	1.06	0.85	5	1.17	0.71	5	0.68	0.21	4	0.56	0.25	3	0.89	0.15	3
ms^{2}t^{6}A	1.26	0.0030	3	1.29	0.055	3	1.05	0.14	3	1.10	0.152	3	Below LOD			Below LOD		
rC	2961.34	10.12	4	2810.40	54.60	4	2975.73	141.92	5	4920.94	1980.27	4	2618.95	114.81	3	2669.50	124.26	4
f^{5}Cm	0.53	0.0070	3	0.45	0.041	3	0.52	0.16	3	0.48	0.043	3	Below LOD			Below LOD		
m^{5}C	79.82	4.48	5	75.74	34.25	5	73.87	45.09	5	82.48	52.39	5	78.71	9.81	4	54.00	5.88	3
m^{3}C	15.97	0.97	5	15.38	8.14	5	13.01	8.09	5	14.72	9.37	5	6.07	0.093	3	6.82	0.83	3
Cm	22.83	0.15	4	22.98	1.50	4	22.31	2.93	4	22.57	2.98	4	23.93	0.52	3	24.75	0.89	3
m^{4}Cm	Below LOD			Below LOD			0.0040	0.00010	2	0.0028	0.0037	3	Below LOD			Below LOD		
ac^{4}C	452.217	1.049	3	490.017	8.344	3	392.916	56.331	3	450.399	52.773	3	29.315	4.232	3	37.086	13.302	4
rU	1033.463	10.965	4	1215.298	275.337	5	1004.554	22.064	4	1087.758	330.329	4	1253.925	50.255	3	1239.284	15.627	3
D	148.565	1.274	4	171.036	18.578	4	136.299	17.306	4	167.397	21.335	4	411.451	73.129	4	325.991	20.831	4
mcm^{5}s^{2}U	2.212	0.009	3	2.372	0.031	3	1.755	0.567	3	1.711	0.224	3	1.860	0.565	3	2.498	0.166	3
mcm^{5}U	0.082	0.001	3	0.061	0.010	3	0.055	0.002	2	0.060	0.005	3	Below LOD			Below LOD		
m^{5}s^{2}U	Below LOD			Below LOD			Below LOD			Below LOD			Below LOD			Below LOD		
ncm^{5}U	6.029	0.356	5	6.252	2.920	5	5.381	2.183	4	7.382	0.860	3	5.575	0.642	4	5.009	0.912	4
Ψ	Below LOD			Below LOD			Below LOD			Below LOD			Below LOD			Below LOD		
m^{5}U	27.103	1.226	5	29.019	11.212	5	25.674	14.164	5	29.273	14.726	5	32.494	6.651	4	27.619	2.409	4
m^{3}U	4.073	0.059	5	3.879	0.315	4	3.961	0.574	5	4.418	0.457	4	3.253	0.344	4	4.649	0.682	4
Um	18.163	0.203	4	17.108	4.750	5	15.232	5.802	5	17.605	7.704	4	10.919	1.265	4	11.463	1.204	4
rG	2800.687	6.871	4	2792.575	55.124	4	3095.621	233.903	4	2373.990	602.027	5	3014.940	212.118	3	3088.306	37.421	3
imG2	Below LOD			Below LOD			0.845	0.118	2	Below LOD			Below LOD			Below LOD		
m^{7}G	49.912	0.229	3	52.551	0.336	3	49.705	3.445	3	55.704	5.703	3	0.708	0.177	3	0.491	0.035	3
m^{2}G	346.754	16.942	5	371.665	147.746	5	309.021	167.354	5	335.753	198.723	5	482.259	29.536	3	304.566	55.945	4
m^{1}G	45.897	2.069	5	50.883	19.491	5	45.923	23.828	5	49.268	27.264	5	67.798	7.230	3	45.115	5.386	4
Gm	33.555	0.336	4	35.066	1.958	4	35.152	5.209	4	38.156	7.441	4	36.315	3.277	4	40.028	3.097	3
m^{2,2}G	88.857	0.300	3	90.543	1.994	3	86.795	6.559	3	97.297	9.854	3	67.791	4.948	3	51.002	5.682	4
I	11.503	0.699	5	18.883	1.976	4	16.770	0.794	3	18.266	1.673	3	15.195	3.256	4	10.735	1.013	4
m^{1}I	0.173	0.002	4	0.198	0.085	5	0.125	0.082	5	0.155	0.057	5	0.414	0.143	3	0.427	0.149	4
Im	0.254	0.004	3	0.237	0.001	2	Below LOD			Below LOD			0.033	0.020	2	Below LOD		

Supplementary Table S3: The average fmol per total injected nucleoside from murine tissue of each ribonucleoside ± the standard deviation (in 10^4 ns). Count (N) represents biological replicates in which the nucleoside was > LOQ.

	Diabetic Aorta			Control Aorta			Diabetes Apex			Control Apex			Diabetes Atria			Control Atria		
**Nucleoside**	**Average**	**Std Dev**	**N**	**Average**	**Std Dev**	**N**	**Average**	**Std Dev**	**N**	**Average**	**Std Dev**	**N**	**Average**	**Std Dev**	**N**	**Average**	**Std Dev**	**N**
rA	1430.58	15.42	3	1744.92	116.23	3	2218.01	115.74	3	2000.94	70.47	3	1808.42	53.93	3	1736.61	112.96	3
m^{6,6}A	0.95	0.43	3	1.43	0.78	3	2.24	0.80	3	4.60	0.94	3	1.71	0.70	3	2.98	0.78	3
m^{6}Am	Below LOD			0.19	0.043	3	0.082	0.030	3	0.098	0.019	3	0.082	0.024	3	0.14	0.014	2
t^{6}A	4.87	1.17	3	4.82	1.31	3	10.00	0.82	3	9.04	0.55	3	10.87	0.28	3	8.70	0.60	3
m^{1}A	Below LOD			Below LOD			16.37	2.50	3	13.10	6.00	3	13.38	3.19	3	6.86	2.81	3
m^{2}A	0.21	0.15	3	0.11	0.040	3	0.020	0.0091	3	0.028	0.015	3	0.027	0.015	3	0.009	0.001	3
Am	2.52	1.57	3	2.27	0.15	3	1.81	0.20	3	2.78	0.95	3	1.87	0.21	3	2.95	0.90	3
m^{6}A	30.89	17.11	3	36.56	9.98	3	87.58	2.72	3	89.36	3.54	3	98.44	3.84	3	89.19	7.85	3
i^{6}A	Below LOD			Below LOD			0.095	0.031	3	0.20	0.064	2	Below LOD			Below LOD		
ms^{2}t^{6}A	Below LOD			Below LOD			0.21	0.021	2	0.2	0.009	2	0.22	0.024	3	Below LOD		
ms^{2}ct^{6}A	Below LOD			Below LOD			0.017	0.01	2	Below LOD			0.017	0.008	2	0.042	0.25	3
rC	4688.35	354.24	3	4362.29	200.83	3	3085.77	109.42	3	3179.38	62.61	3	3330.77	77.25	3	3516.55	173.10	3
f^{5}C	Below LOD			Below LOD			0.079	0.0021	3	0.076	0.0016	3	0.050	0.004	2	Below LOD		
m^{5}C	30.63	21.96	3	34.86	10.34	3	78.30	8.71	3	80.35	5.39	3	91.09	4.07	3	73.91	4.38	3
m^{3}C	4.86	2.24	3	5.46	1.28	3	9.99	1.43	3	10.64	0.89	3	11.16	0.28	3	9.39	0.42	3
Cm	9.35	4.96	3	10.49	1.75	3	14.06	0.79	3	15.13	1.24	3	17.91	0.82	3	16.15	1.78	3
m^{4}Cm	0.36	0.13	3	0.35	0.054	2	Below LOD			0.093	0.012	2	0.079	0.021	2	Below LOD		
ac^{4}C	2.50	0.65	3	5.95	5.87	3	3.24	1.16	3	3.64	0.25	3	2.01	0.55	3	3.68	2.24	2
ac^{4}Cm	0.16	0.064	2	0.50	0.109	2	Below LOD			Below LOD			0.071	0.014	2	0.17	0.025	3
rU	2129.87	462.33	3	1893.93	59.63	3	1446.61	11.96	3	1458.00	6.097	3	1364.57	18.14	3	1594.24	180.17	3
D	41.52	33.17	2	176.64	196.71	3	98.04	37.04	3	130.85	29.19	3	85.20	29.15	3	151.31	100.32	2
mcm^{5}s^{2}U	17.10	3.49	3	6.01	4.71	3	2.09	0.87	3	2.69	1.54	3	0.83	0.26	2	3.81	2.49	3
mcm^{5}U	Below LOD			Below LOD			0.020	0.0010	2	0.021	0.0016	3	0.029	0.001	2	Below LOD		
ncm^{5}U	4.25	0.63	2	4.04	1.20	3	5.06	0.27	3	4.99	0.29	3	6.16	0.31	3	5.11	0.63	3
Ψ	265.46	97.92	2	Below LOD			61.25	13.89	3	55.26	11.63	3	63.80	15.51	3	115.97	39.79	3
m^{5}U	17.32	8.95	3	17.02	4.76	3	40.94	0.99	3	38.91	1.53	3	44.64	1.06	3	37.66	4.93	3
m^{3}U	Below LOD			Below LOD			2.98	0.30	3	2.68	0.61	3	3.09	0.35	3	2.16	0.95	3
Um	3.49	0.48	2	6.17	0.43	3	7.70	2.65	3	6.88	3.86	3	3.17	0.44	3	1.95	0.48	3
rG	1086.70	590.00	3	1244.49	133.53	3	1969.18	26.03	3	2102.01	87.41	3	2136.90	53.76	3	1926.42	216.51	3
imG2	4.61	1.46	3	3.15	0.92	3	0.57	0.14	3	0.50	0.040	3	0.40	0.12	3	0.48	0.16	3
m^{7}G	28.83	8.81	2	21.82	7.01	3	42.53	0.99	3	41.69	2.25	3	53.40	0.54	3	44.35	8.71	3
m^{2}G	232.60	131.01	3	264.72	61.65	3	620.64	22.42	3	572.54	25.64	3	649.94	39.71	3	536.36	69.67	3
m^{1}G	26.22	12.2	2	20.41	7.36	3	61.14	1.14	3	58.32	3.17	3	65.17	1.23	3	51.09	5.26	3
Gm	16.59	9.81	3	17.44	2.01	3	24.83	1.60	3	28.27	2.83	3	30.87	1.97	3	29.92	4.05	3
m^{2,2}G	38.50	20.69	3	36.67	6.84	3	77.17	1.80	3	74.60	1.73	3	89.92	0.92	3	73.54	7.64	3
I	Below LOD			Below LOD			10.99	0.60	3	11.82	0.40	3	13.58	0.62	3	9.47	4.26	3
m^{1}I	0.15	0.10	3	0.23	0.051	3	0.42	0.017	3	0.44	0.0054	3	0.51	0.0041	3	0.41	0.047	3

Supplementary Table S4: Comparison of detected tRNA modifications in mouse tissue/cells with tRNA modifications detected in our work

			(Richter, F. et al. 2021)[1]	(Richter, F. et al. 2021)[1]	(Lene Songe-Møller et al. 2010)[2]	Modomics: 2021 update[3]	(Endres, L. et al. 2015)[4]	(Yan, M. et al 2013)[5]	(Li, A. et al. 2022)[6]	Our work
**Nucleoside**	**Symbol**	**MRM**	**Mouse liver tissue**	**Mouse brain tissue**	**Mouse liver/ testis/ brain**		**Murine embryonic fibroblasts**	**Mouse liver (male)**	**Mouse brain/heart/ liver**	**Heart**
2-methylthio-N^6-isopentenyladenosine	ms^{2}i^{6}A	**382>250**	x	x						
N^6,N^6,2'-O-trimethyladenosine	m^{6,6}Am	**310>164**								
N^6,N^6-dimethyladenosine	m^{6,6}A	**296>164**	x*	x*						x
N^6-formyladenosine	f^{6}A	**296>164**								
N^1-2'-O-dimethyladenosine	m^{1}Am	**296>150**								
N^6-2'-O-dimethyladenosine	m^{6}Am	**296>150**								x
N^6-(N-threonylcarbonyl)adenosine	t^{6}A	**413>281**	x	x		x				x
N^1-methyladenosine	m^{1}A	**282>150**	x	x		x		x		x
C^2-methyladenosine	m^{2}A	**282>150**	x	x						x
2'-O-methyladenosine	Am	**282>136**	x*	x*				x		x
8-methyladenosine	m^{8}A	**282>150**								
N^6-methyladenosine	m^{6}A	**282>150**	x	x				x		x
2-methylthio-N^6-methyladenosine	ms^{2}m^{6}A	**328>196**								
N^6-(3-methyl-2-butenyl)adenosine	i^{6}A	**336>204**	x	x		x		x		x
2-methylthio-N^6-threonylcarbamoyladenosine	ms^{2}t^{6}A	**459>327**								x
2-methylthio-N^6-(cis-hydroxyisopentenyl)adenosine	ms^{2}io^{6}A	**398>266**								
N^6-(cis-hydroxyisopentenyl) adenosine	io^{6}A	**352>220**								
Cyclic N^6-threonylcarbamoyladenoside	ct^{6}A	**395>263**								
Cyclic 2-methylthio-N^6-threonylcarbamoyladenoside	ms^{2}ct^{6}A	**441>309**								x
N^6-methyl-N^6-threonylcarbamoyl adenosine	m^{6}t^{6}A	**427>295**								
5-formyl-2'-O-methylcytosine	f^{5}Cm	**286>140**								
5-formylcytosine	f^{5}C	**272>140**							x	x
5-hydroxymethylcytosine	hm^{5}C	**274>142**						x		
5-methylcytosine	m^{5}C	**258>126**	x	x		x		x		x
N^4-methylcytidine	m^{4}C	**258>126**								
N^3-methylcytidine	m^{3}C	**258>126**	x	x				x		x
2-thiocytidine	s^{2}C	**260>128**								
2'-O-methylcytidine	Cm	**258>112**	x	x		x		x		x
N^4-methyl-2'-O-methylcytidine	m^{4}Cm	**272>126**								x
N-acetylcytidine	ac^{4}C	**286>154**						x		x
N^4-acetyl-2'-O-methylcytidine	ac^{4}Cm	**300>154**								x
Dihydrouridine	D	**247>115**	x	x		x				x

5-methyoxycarbonylmethyl-2-thiouridine	mcm^{5}s^{2}U	**333>201**			x		x			x
5-methyl-2-thiouridine	m^{5}s^{2}U	**275>143**								
5-methyoxycarbonylmethyluridine	mcm^{5}U	**317>185**	x	x	x		x			x
5-hydroxyuridine	ho^{5}U	**261>129**								
5-carbamoylmethyluridine	ncm^{5}U	**302>170**			x					x
1-methylpseudouridine	m$^1\Psi$	**259>223**								
Pseudouridine	Ψ	**245>209**	x	x		x		x		x
3-methylpseudouridine	m$^3\Psi$	**259>223**								
5-methyluridine	m^{5}U	**259>127**	x	x				x		x
Uridine-5-oxyacetic acid	cmo^{5}U	**319>187**								
2-thiouridine	s^{2}U	**261>129**								
4-thiouridine	s^{4}U	**261>129**								
5-methoxyuridine	mo^{5}U	**275>143**								
3'-O-methyluridine	NA	**259>113**								
N^3-methyluridine	m^{3}U	**259>127**	x*	x*				x		x
2'-O-methyluridine	Um	**259>113**	x	x		x		x		x
N^{2,7}-dimethylguanosine	m^{2,7}G	**312>180**								
Isowyosine	imG2	**336>204**								x
7-methylguanosine	m^{7}G	**298>166**	x	x		x		x		x
N^2-methylguanosine	m^{2}G	**298>166**	x	x		x		x		x
N^1-methylguanosine	m^{1}G	**298>166**	x	x		x		x		x
2'-O-methylguanosine	Gm	**298>152**	x	x		x		x		x
N^2,N^2-dimethylguanosine	m^{2,2}G	**312>180**	x	x		x		x		x
Queuosine	Q	**410>295**	x	x						
Inosine	I	**269>137**	x	x		x		x		x
N^1-methylinosine	m^{1}I	**283>151**								x
2'-O-methylinosine	Im	**283>137**						x		
2'-O-methylthymidine	Tm	**273>127**	x	x						
5-methoxycarbonylmethyl-2'-O-methyluridine	mcm^{5}Um	**331>185**			x					
5,2'-O-dimethyluridine	m^{5}Um	**273>127**				x	x	x		
Wybutosine	yW	**509>377**				x				
5-carboxymethyluridine	cm^{5}U	**303>171**			x					
5-caramoylmethyl-2-thiouridine	ncm^{5}s^{2}U	**318>186**			x					
N^{2,2,7}-trimethylguanosine	m^{2,2,7}G	**326>194**						x		
N^5-methyl-2'-O-methylcytidine	m^{5}Cm	**272>126**						x		
* indicates presence in tRNA due to potential ribosomal RNA (rRNA) degradation										

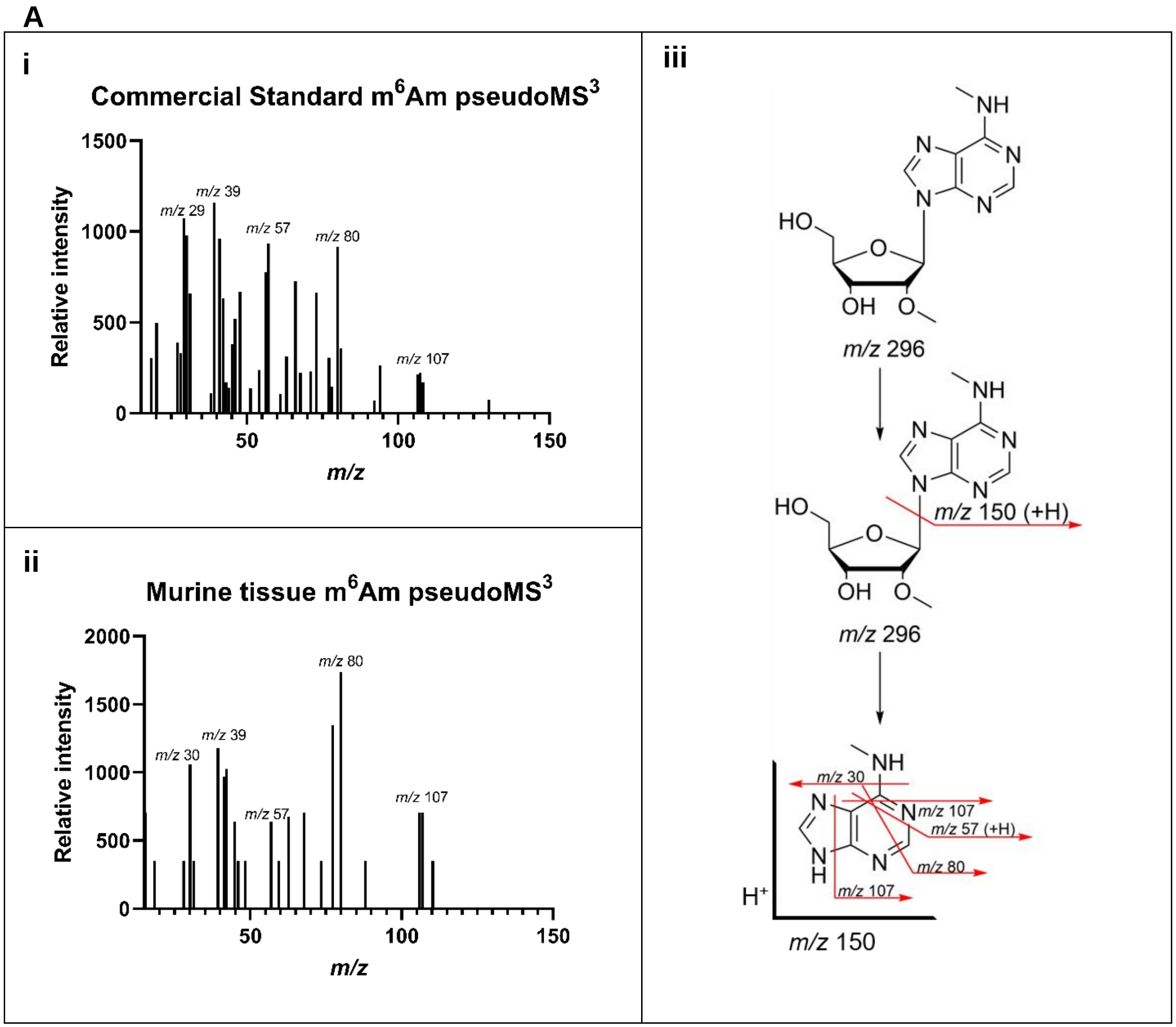
A
i
Commercial Standard m6Am pseudoMS3
Relative intensity
m/z
m/z 29
m/z 39
m/z 57
m/z 80
m/z 107
ii
Murine tissue m6Am pseudoMS3
Relative intensity
m/z
m/z 30
m/z 39
m/z 57
m/z 80
m/z 107
iii
m/z 296
m/z 150 (+H)
m/z 296
m/z 30
m/z 107
m/z 57 (+H)
m/z 80
m/z 107
m/z 150

B

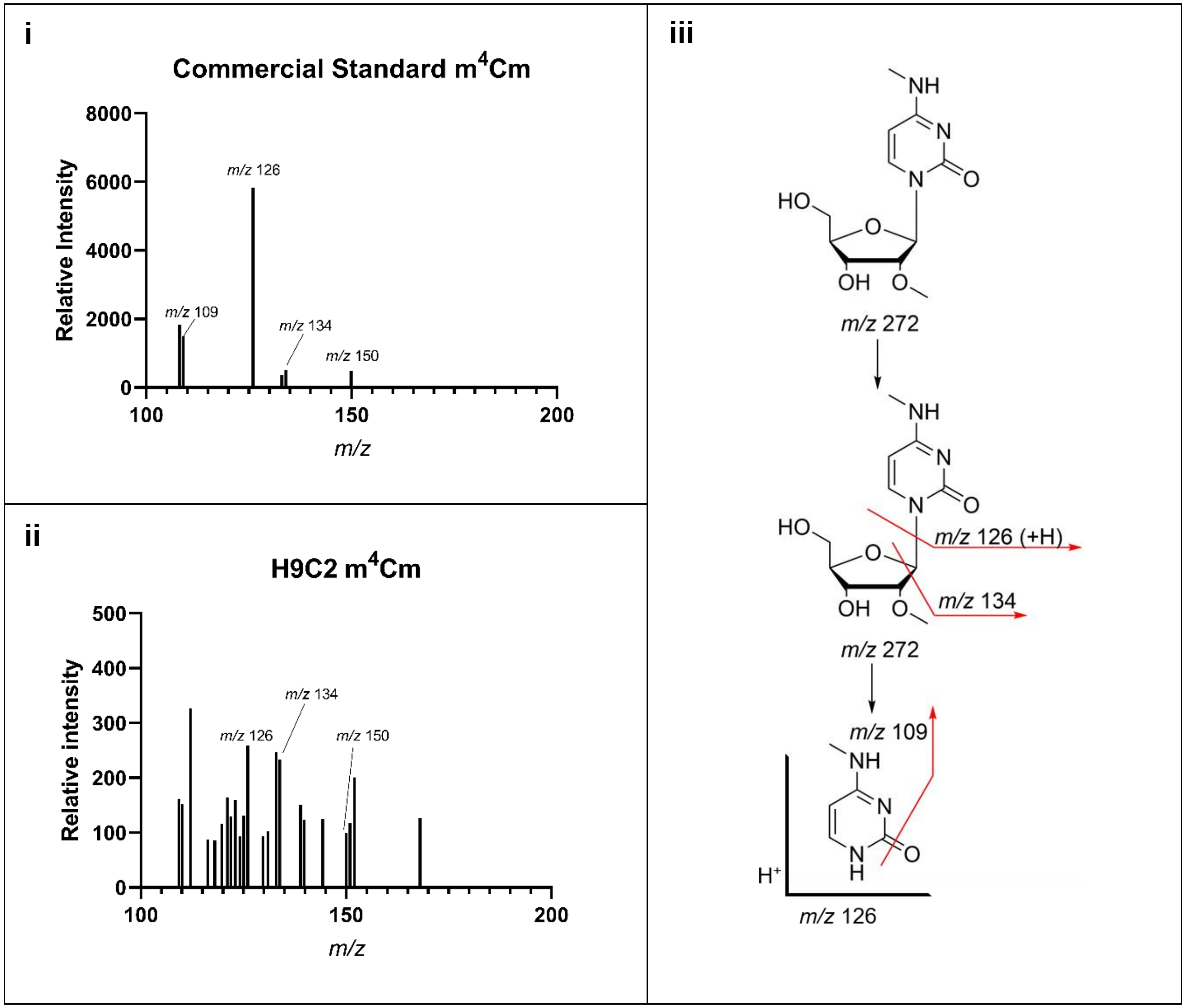
i
Commercial Standard m4Cm
Relative Intensity
8000
6000
4000
2000
0
100
150
200
m/z
m/z 126
m/z 109
m/z 134
m/z 150
ii
H9C2 m4Cm
Relative intensity
500
400
300
200
100
0
100
150
200
m/z
m/z 126
m/z 134
m/z 150
iii
NH
N
O
N
HO
O
OH
O
m/z 272
m/z 126 (+H)
m/z 134
m/z 109
H+
m/z 126

C

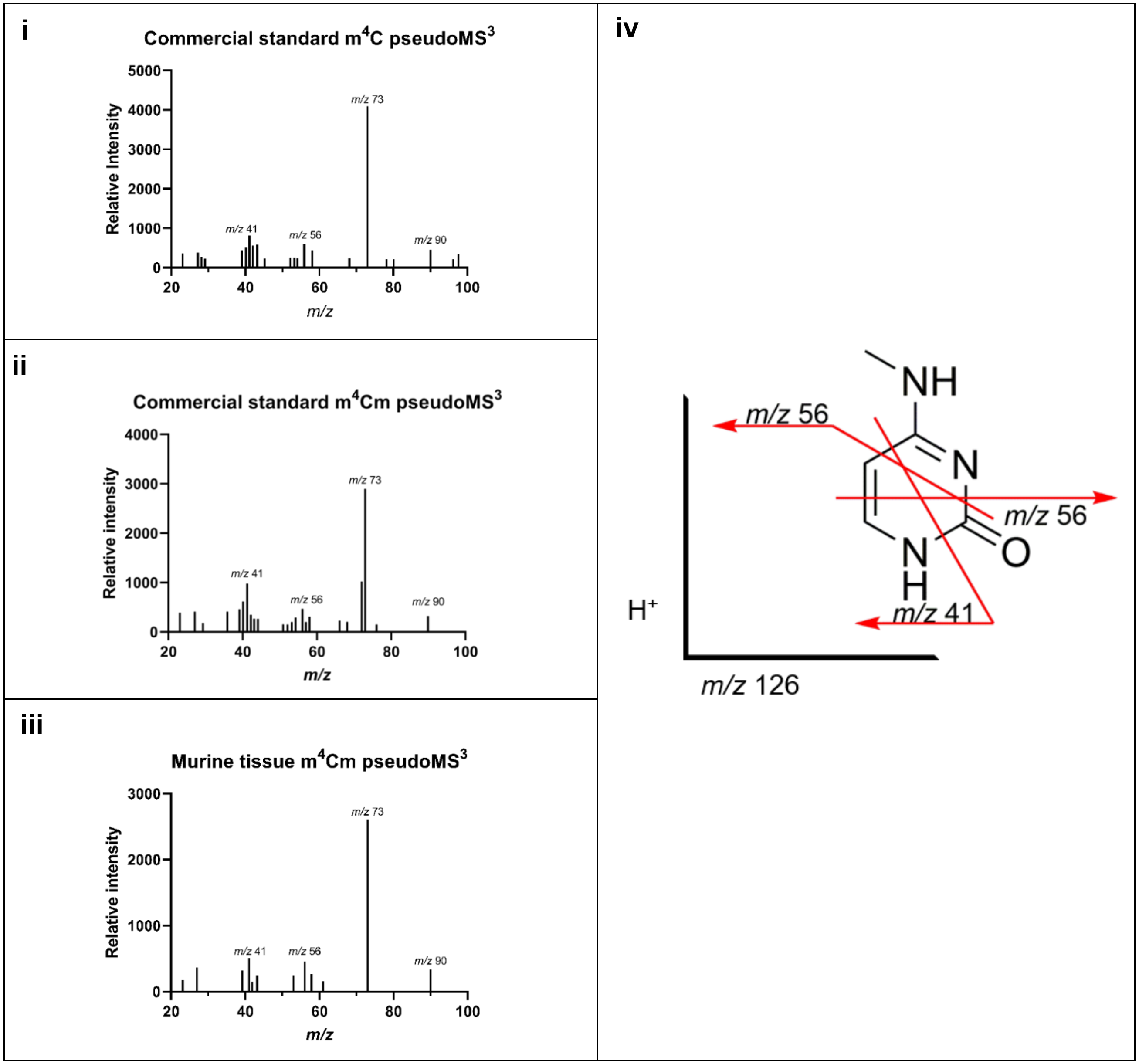

i
Commercial standard m4C pseudoMS3
Relative Intensity
5000
4000
3000
2000
1000
0
20
40
60
80
100
m/z
m/z 41
m/z 56
m/z 73
m/z 90
ii
Commercial standard m4Cm pseudoMS3
Relative intensity
4000
3000
2000
1000
0
20
40
60
80
100
m/z
m/z 41
m/z 56
m/z 73
m/z 90
iii
Murine tissue m4Cm pseudoMS3
Relative intensity
3000
2000
1000
0
20
40
60
80
100
m/z
m/z 41
m/z 56
m/z 73
m/z 90
iv
NH
N
N
H
O
H+
m/z 56
m/z 56
m/z 41
m/z 126

D

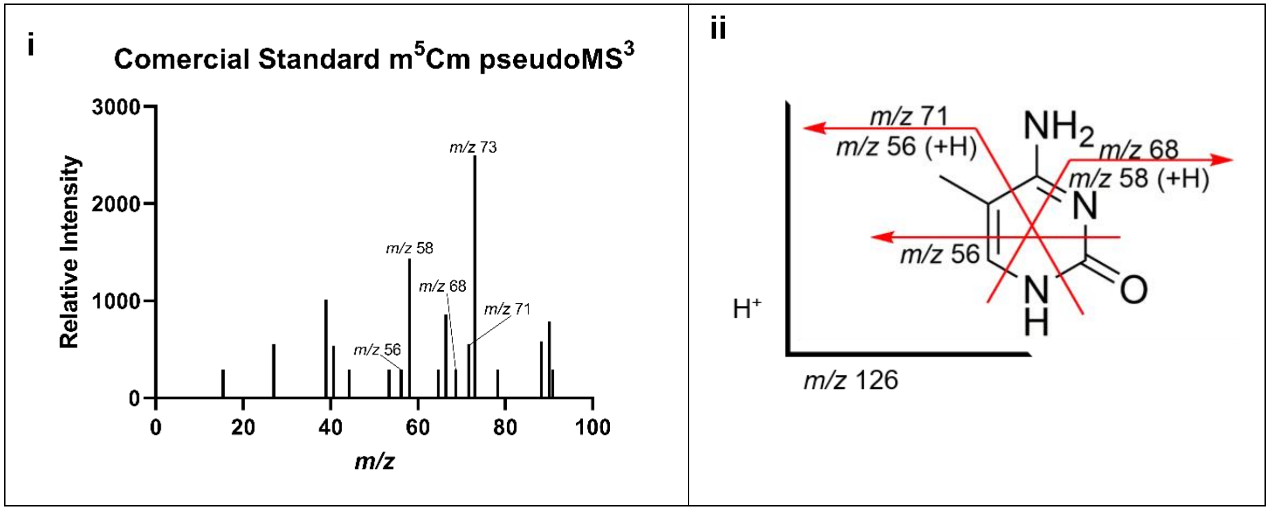

i
Comercial Standard m5Cm pseudoMS3
Relative Intensity
3000
2000
1000
0
0
20
40
60
80
100
m/z
m/z 73
m/z 58
m/z 68
m/z 71
m/z 56
ii
m/z 71
m/z 56 (+H)
NH2
m/z 68
m/z 58 (+H)
N
m/z 56
N
H
O
H+
m/z 126

E

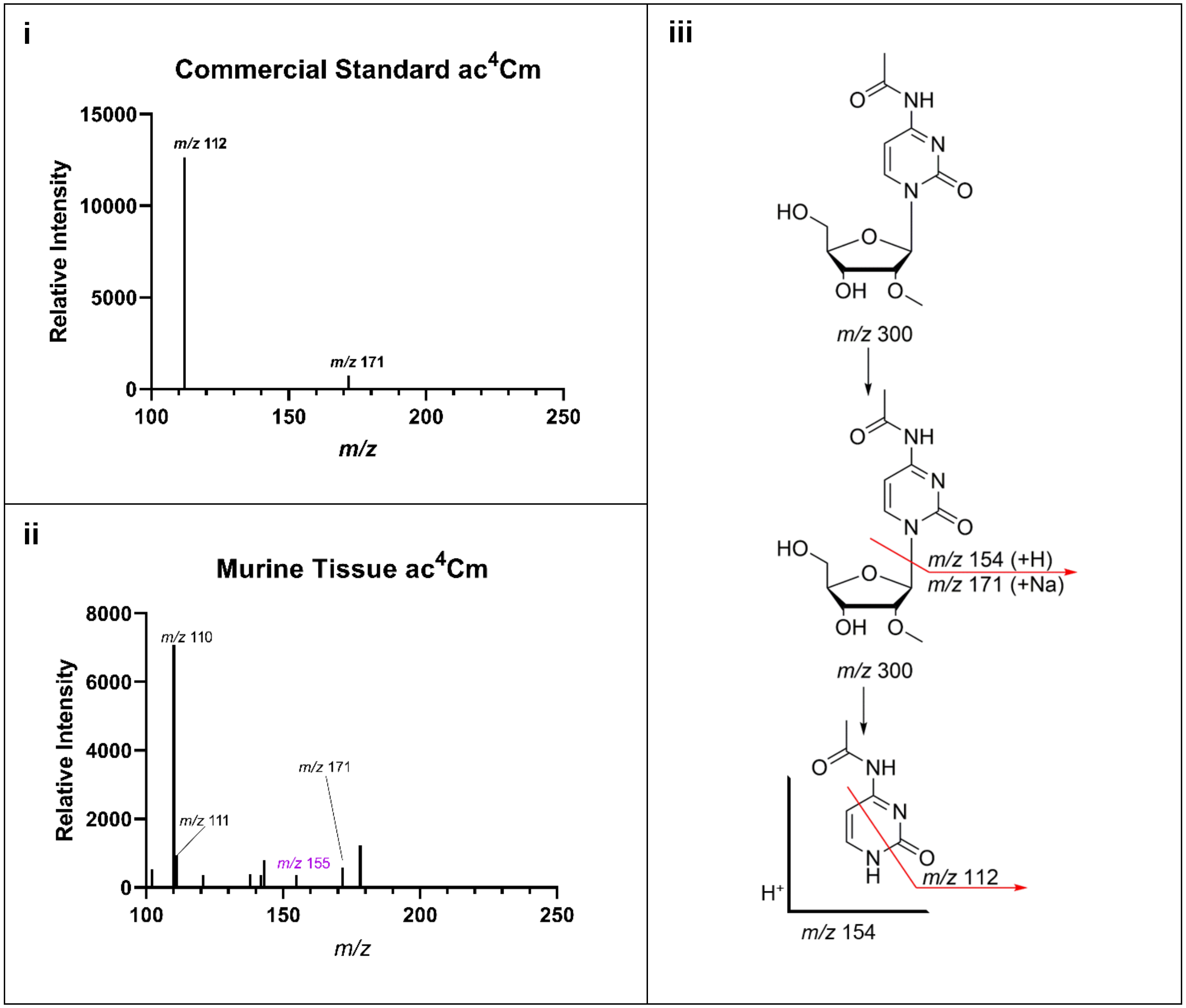

i
Commercial Standard ac4Cm
Relative Intensity
15000
10000
5000
0
m/z 112
m/z 171
100
150
200
250
m/z
ii
Murine Tissue ac4Cm
Relative Intensity
8000
6000
4000
2000
0
m/z 110
m/z 111
m/z 155
m/z 171
100
150
200
250
m/z
iii
O
NH
N
N
O
HO
O
OH
O
m/z 300
m/z 154 (+H)
m/z 171 (+Na)
m/z 300
H+
N
H
m/z 112
m/z 154

F

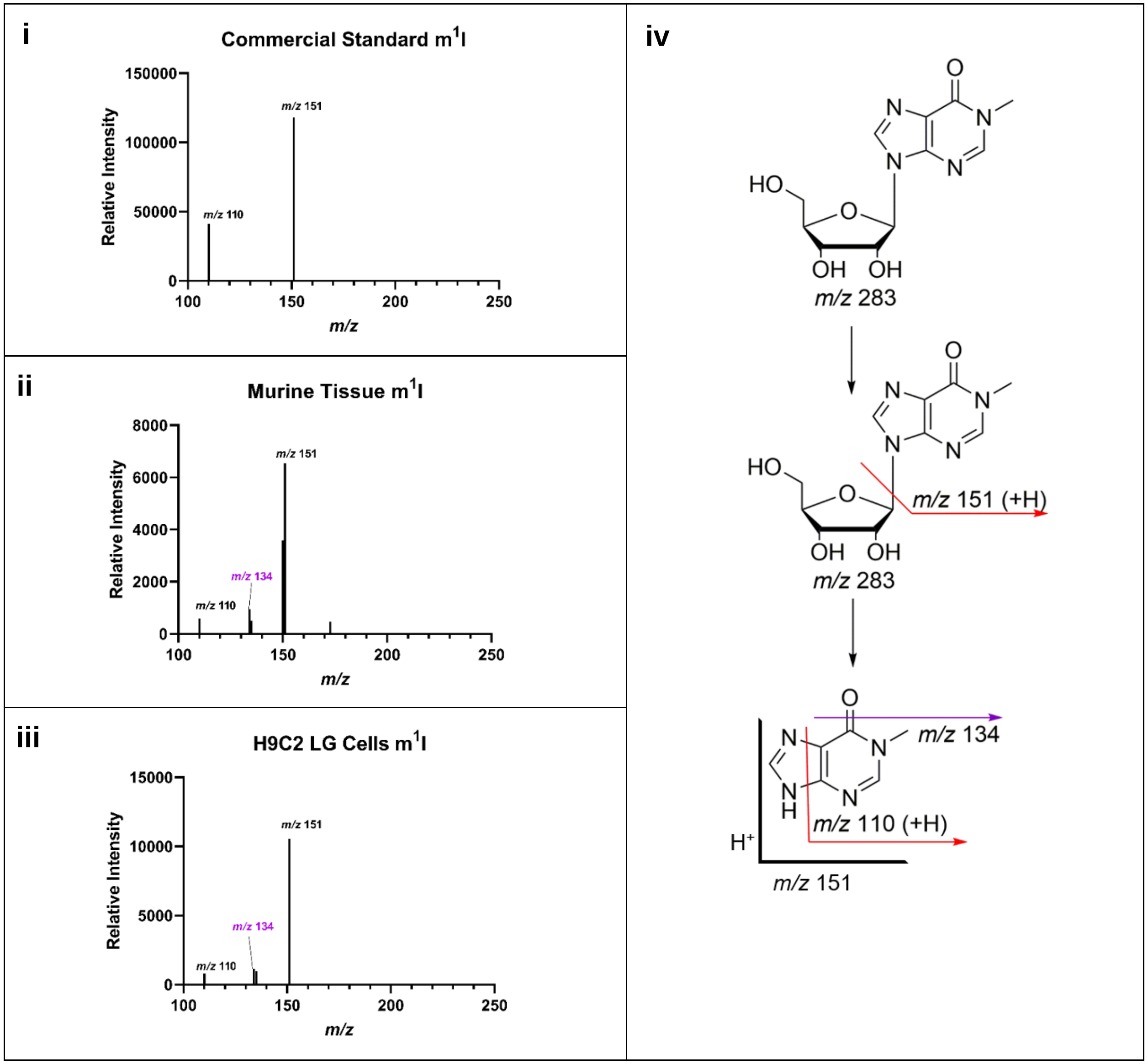
i
Commercial Standard m1I
Relative Intensity
150000
100000
50000
0
m/z 151
m/z 110
100
150
200
250
m/z
ii
Murine Tissue m1I
Relative Intensity
8000
6000
4000
2000
0
m/z 151
m/z 134
m/z 110
100
150
200
250
m/z
iii
H9C2 LG Cells m1I
Relative Intensity
15000
10000
5000
0
m/z 151
m/z 134
m/z 110
100
150
200
250
m/z
iv
m/z 283
m/z 151 (+H)
m/z 283
m/z 134
m/z 110 (+H)
H+
m/z 151

G

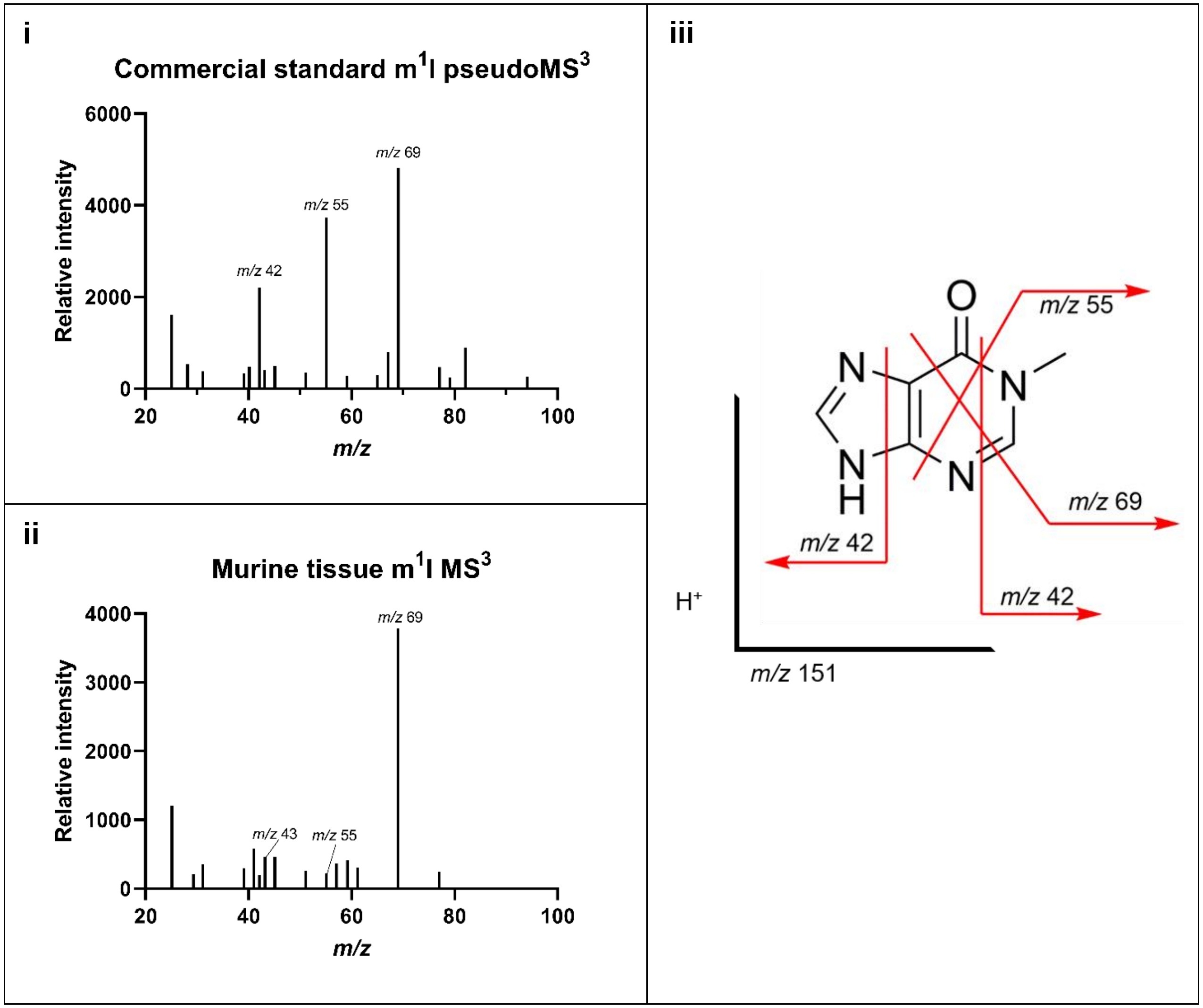
i
Commercial standard m1I pseudoMS3
Relative intensity
m/z
m/z 42
m/z 55
m/z 69
6000
4000
2000
0
20
40
60
80
100
ii
Murine tissue m1I MS3
Relative intensity
m/z
m/z 43
m/z 55
m/z 69
4000
3000
2000
1000
0
20
40
60
80
100
iii
m/z 55
m/z 69
m/z 42
m/z 42
H+
m/z 151

H

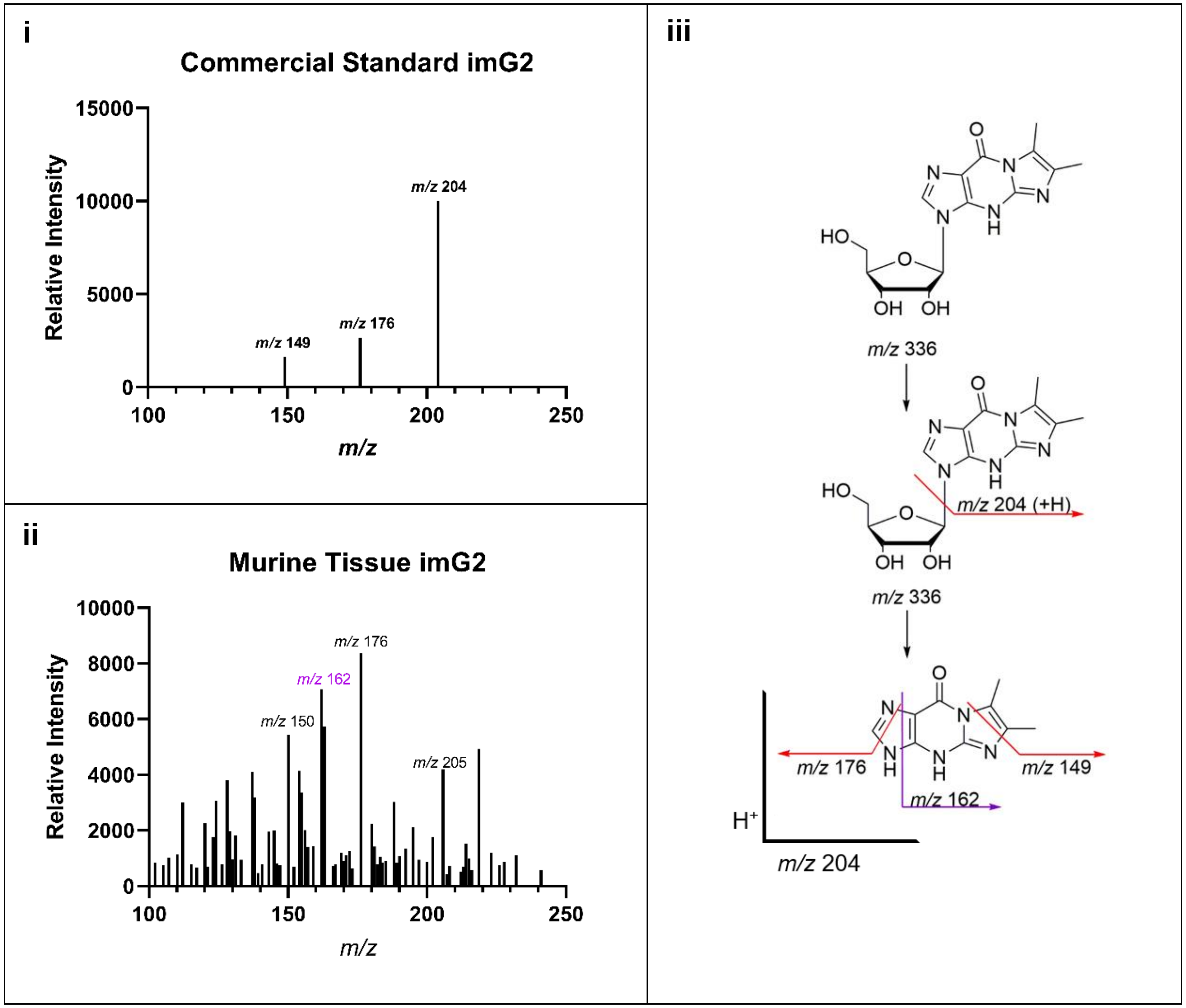

i
Commercial Standard imG2
Relative Intensity
15000
10000
5000
0
100
150
200
250
m/z
m/z 149
m/z 176
m/z 204
ii
Murine Tissue imG2
Relative Intensity
10000
8000
6000
4000
2000
0
100
150
200
250
m/z
m/z 150
m/z 162
m/z 176
m/z 205
iii
m/z 336
m/z 204 (+H)
m/z 336
m/z 176
m/z 162
m/z 149
H+
m/z 204

I

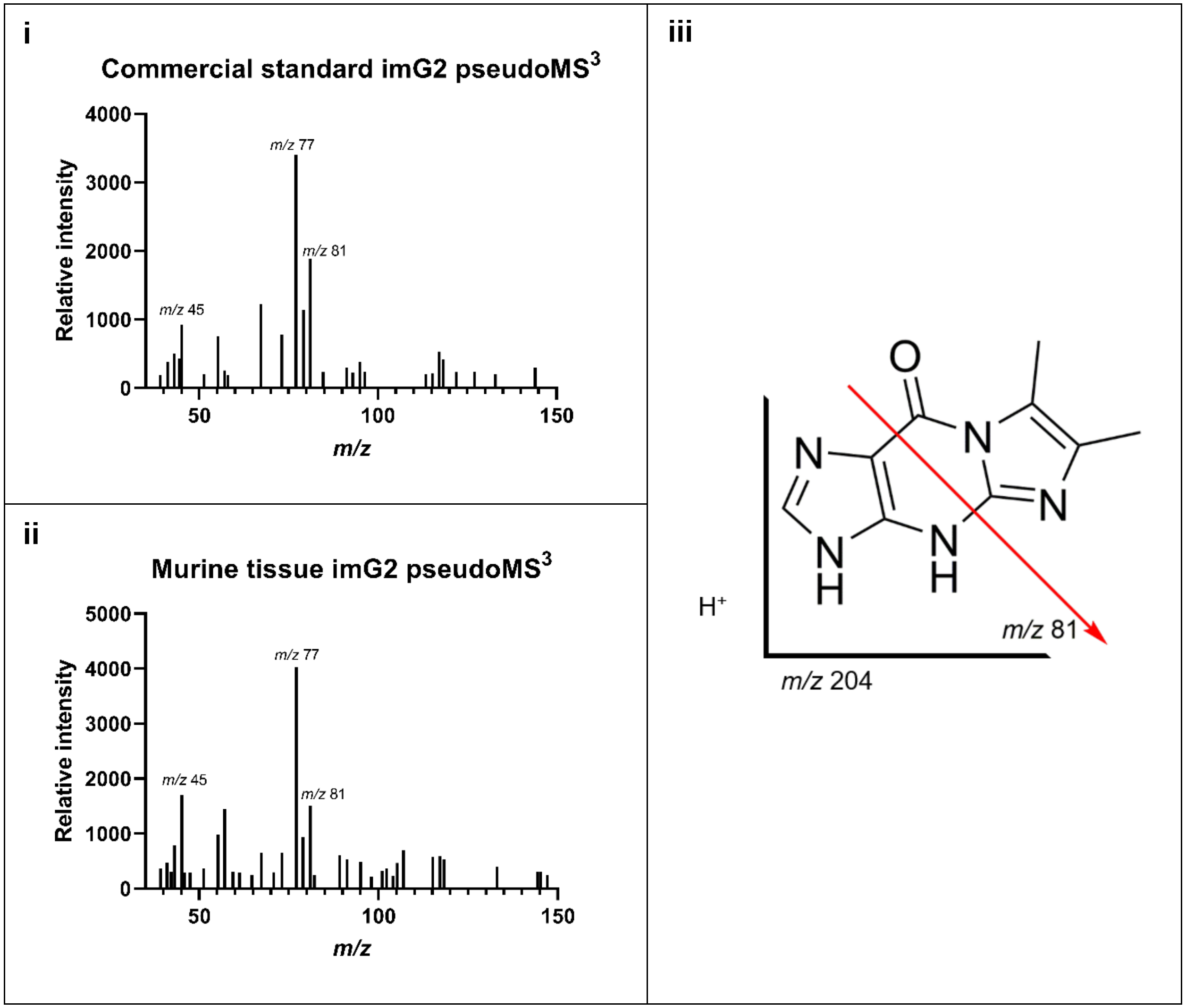
i
Commercial standard imG2 pseudoMS3
Relative intensity
m/z 77
m/z 81
m/z 45
4000
3000
2000
1000
0
50
100
150
m/z
ii
Murine tissue imG2 pseudoMS3
Relative intensity
m/z 77
m/z 45
m/z 81
5000
4000
3000
2000
1000
0
50
100
150
m/z
iii
O
N
N
N
N
H
N
H
H+
m/z 81
m/z 204

Supplementary Figure S8: Mass spectra fragmentation of RNA modifications detected in mammalian *in vitro* cells or murine heart tissue. Comparisons to MS fragmentation of commercially available standards in positive ion mode. Purple ion masses indicate those only identified in endogenous samples but not commercial standards. A) MS/MS m^6Am precursor ion *m/z* 150; B) MS/MS m^4Cm precursor ion *m/z* 272; C) MS/MS m^4Cm precursor ion *m/z* 126; D) MS/MS m^5Cm precursor ion *m/z* 126; E) MS/MS ac^4Cm precursor ion *m/z* 300; F) MS/MS m^1I precursor ion *m/z* 294; G) MS/MS m^1I precursor ion *m/z* 151; H) MS/MS imG2 precursor ion *m/z* 336; I) MS/MS imG2 precursor ion *m/z* 204.

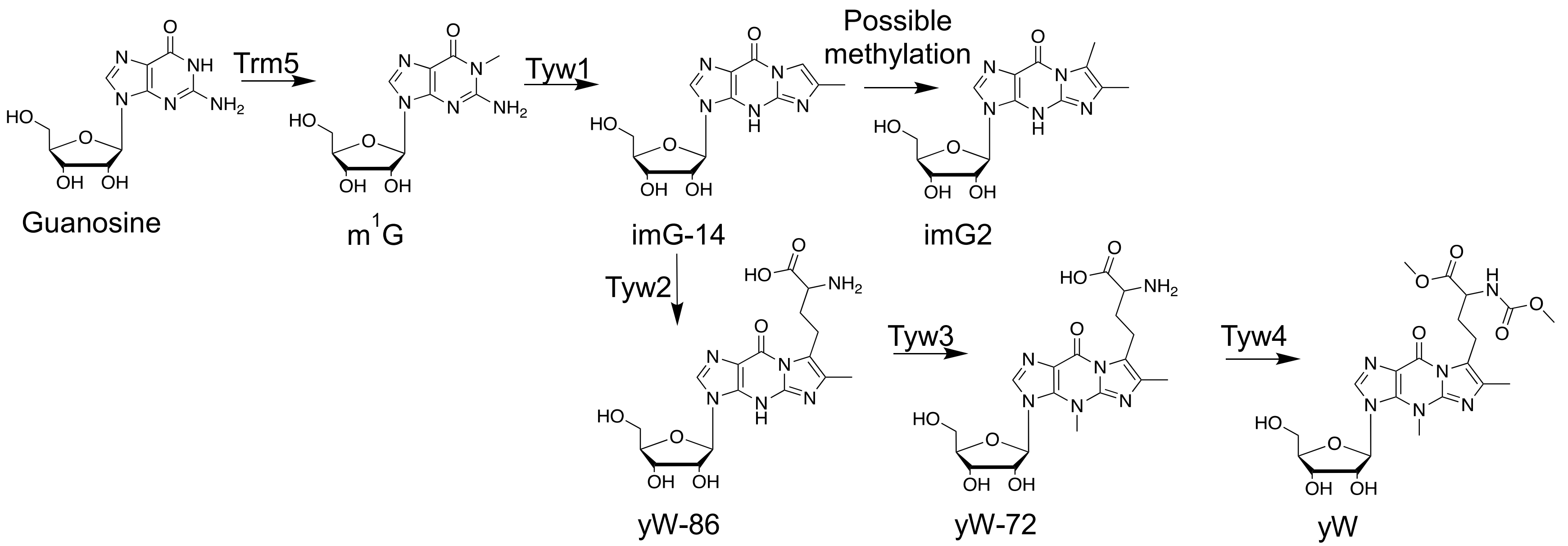

Supplementary Figure S9: Biosynthesis of wyosine derivatives in eukaryotes; including the possible biosynthesis of imG2 in eukaryotes by Trm5.

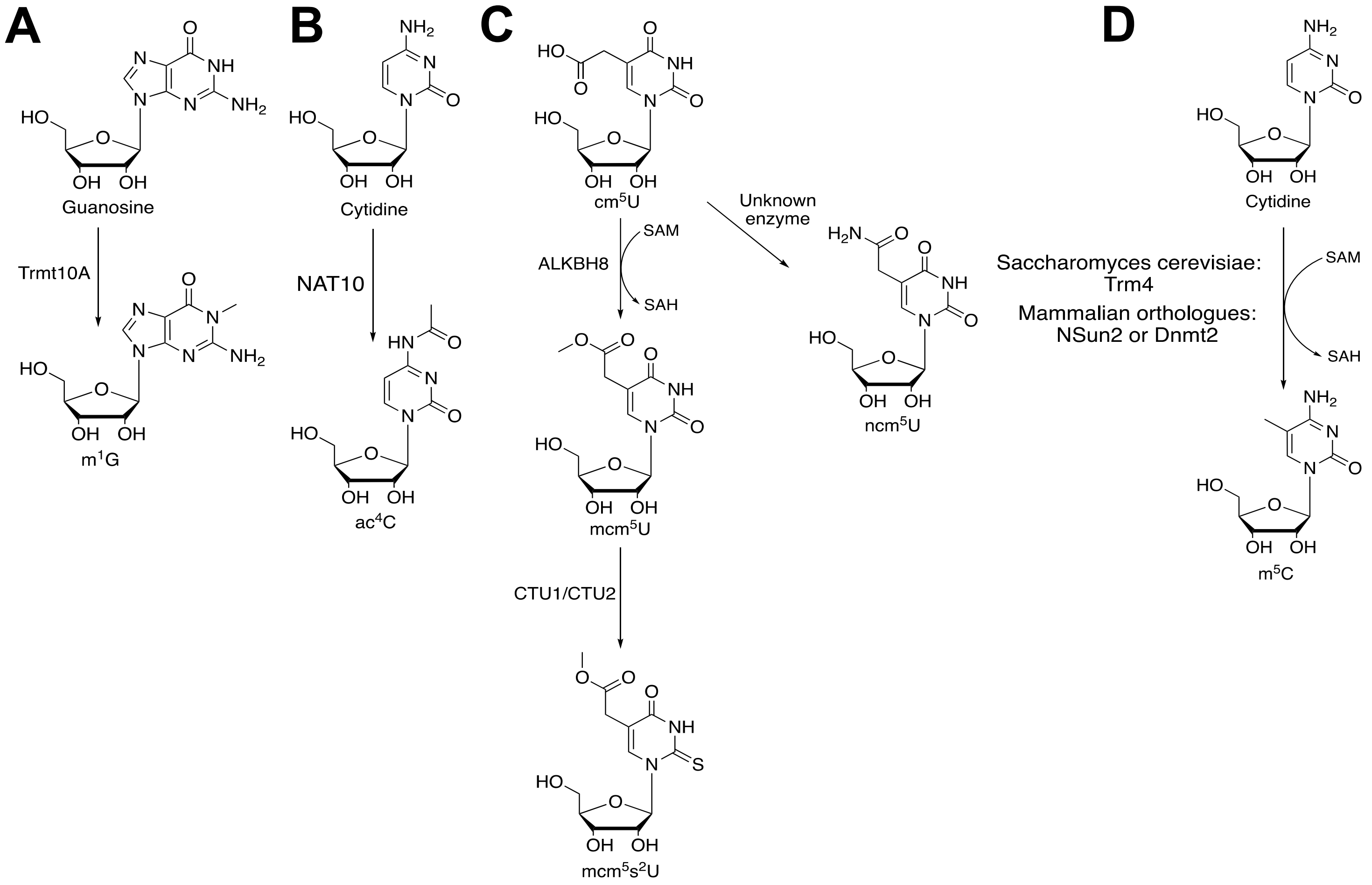

Supplementary Figure S10: tRNA modification biosynthetic schemes, including probable/confirmed tRNA modifying enzymes; A) m^1G B) ac^4C; C) ncm^5U, mcm^5U, and mcm^5s^2U; D) m^5C.

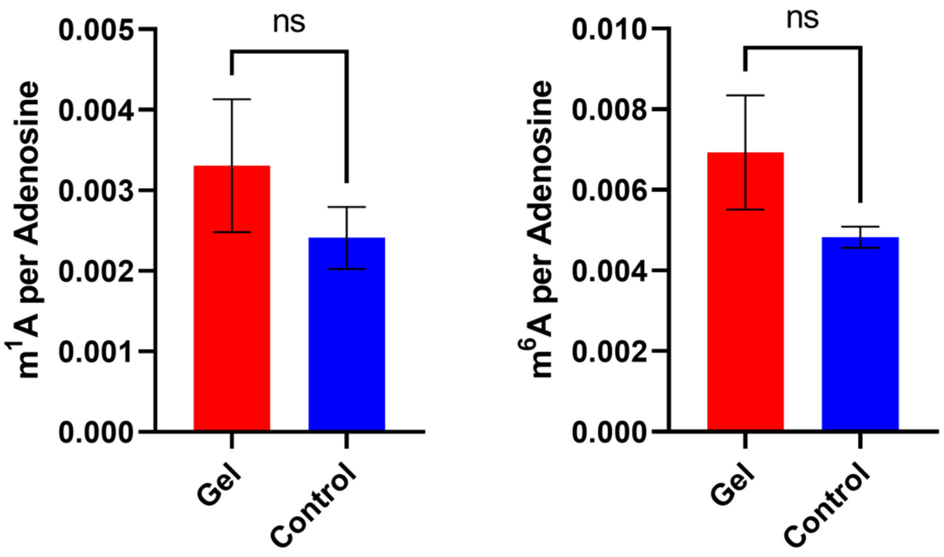

Supplementary Figure S11: Dimroth rearrangement control experiment. Levels of m^1A and m^6A were quantified by LC-MS/MS from Torula yeast RNA analyzed after isolation from PAGE (red) and analyzed without PAGE preparation (blue). A Welch's t-test was performed to test significance, (n=4).

Supporting information references:

[1] Richter, F. et al. (2021). RNA marker modifications reveal the necessity for rigorous preparation protocols to avoid artifacts in epitranscriptomic analysis. Nucleic Acids Res

[2] Songe-Moller, L. et al. (2010). Mammalian ALKBH8 possesses tRNA methyltransferase activity required for the biogenesis of multiple wobble uridine modifications implicated in translational decoding. Mol Cell Biol 30, 1814-27.

[3] Boccaletto, P. et al. (2022). MODOMICS: a database of RNA modification pathways. 2021 update. Nucleic Acids Res 50, D231-D235.

[4] Endres, L. et al. (2015). Alkbh8 Regulates Selenocysteine-Protein Expression to Protect against Reactive Oxygen Species Damage. PLoS One 10, e0131335.

[5] Yan, M. et al. (2013). A high-throughput quantitative approach reveals more small RNA modifications in mouse liver and their correlation with diabetes. Anal Chem 85, 12173-81.

[6] Li, A., Sun, X., Arguello, A.E. and Kleiner, R.E. (2022). Chemical Method to Sequence 5-Formylcytosine on RNA. ACS Chem Biol 17, 503-508.
